# STX-478, a Mutant-Selective, Allosteric PI3Kα Inhibitor Spares Metabolic Dysfunction and Improves Therapeutic Response in PI3Kα-Mutant Xenografts

**DOI:** 10.1158/2159-8290.CD-23-0396

**Published:** 2023-08-25

**Authors:** Leonard Buckbinder, David J. St. Jean, Trang Tieu, Brendon Ladd, Brendan Hilbert, Weixue Wang, Jacob T. Alltucker, Samantha Manimala, Gregory V. Kryukov, Natasja Brooijmans, Gregory Dowdell, Philip Jonsson, Michael Huff, Angel Guzman-Perez, Erica L. Jackson, Marcus D. Goncalves, Darrin D. Stuart

**Affiliations:** 1 Research and Development, Scorpion Therapeutics, Boston, Massachusetts.; 2 Department of Biology, Scorpion Therapeutics, South San Francisco, California.; 3 Division of Endocrinology, Department of Medicine, Weill Cornell Medicine, New York, New York.

## Abstract

Phosphoinositide 3-kinase α (*PIK3CA*) is one of the most mutated genes across cancers, especially breast, gynecologic, and head and neck squamous cell carcinoma tumors. Mutations occur throughout the gene, but hotspot mutations in the helical and kinase domains predominate. The therapeutic benefit of isoform-selective PI3Kα inhibition was established with alpelisib, which displays equipotent activity against the wild-type and mutant enzyme. Inhibition of wild-type PI3Kα is associated with severe hyperglycemia and rash, which limits alpelisib use and suggests that selectively targeting mutant PI3Kα could reduce toxicity and improve efficacy. Here we describe STX-478, an allosteric PI3Kα inhibitor that selectively targets prevalent PI3Kα helical- and kinase-domain mutant tumors. STX-478 demonstrated robust efficacy in human tumor xenografts without causing the metabolic dysfunction observed with alpelisib. Combining STX-478 with fulvestrant and/or cyclin-dependent kinase 4/6 inhibitors was well tolerated and provided robust and durable tumor regression in ER^+^HER2^−^ xenograft tumor models.

**Significance:**

These preclinical data demonstrate that the mutant-selective, allosteric PI3Kα inhibitor STX-478 provides robust efficacy while avoiding the metabolic dysfunction associated with the nonselective inhibitor alpelisib. Our results support the ongoing clinical evaluation of STX-478 in PI3Kα-mutated cancers, which is expected to expand the therapeutic window and mitigate counterregulatory insulin release.

*See related commentary by Kearney and Vasan, p. 2313.*

*This article is featured in Selected Articles from This Issue, p. 2293*

## Teaser

The preclinical profile of STX-478, a mutant-selective, allosteric PI3Kα inhibitor, demonstrates its potential to expand the limited therapeutic window of approved inhibitors and significantly improve outcomes in patients with PI3Kα-mutant cancers.

## INTRODUCTION

The phosphoinositide 3-kinase (PI3K)/AKT axis is pivotal in the development of cancer (1, 2), with oncogenic genetic alterations in PI3Kα occurring in approximately 14% of cancers (3, 4). These mutations are found throughout the *PIK3CA* gene but are highly enriched at hotspot amino acid sites in the helical (E542K, E545K) and kinase (H1047R/L) domains (3, 5), though the frequency of PI3Kα hotspot mutations varies across cancers (6). PI3Kα mutations are most prevalent in breast cancer, occurring in about 36% of patients, of which approximately 28% are helical-domain mutations and 40% are kinase-domain mutations (7). Mutant PI3Kα is also a common oncogenic driver of other difficult-to-treat cancers, including gastric cancer (15%), colon cancer (25%), head and neck squamous cell carcinoma (HNSCC; 13%), and uterine cancer (45%; refs. 3, 8, 9).

The clinical benefit of isoform-selective inhibition of PI3Kα has been demonstrated with alpelisib in PI3Kα-mutant cancers. Alpelisib is an orthosteric inhibitor that shows equipotent inhibition of both wild-type (WT) and mutant forms of PI3Kα. In a phase III trial, alpelisib demonstrated therapeutic activity in PI3Kα-mutant, ER^+^HER2^−^ metastatic breast cancer when used in combination with fulvestrant, a selective estrogen receptor degrader. At a median follow-up of 20 months, this combination demonstrated an almost 2-fold increase in progression-free survival time to 11.0 months compared with 5.7 months for fulvestrant alone in this patient population (*P* < 0.001; ref. 10).

In normal tissue, the PI3K/AKT signal transduction pathway is essential for insulin-dependent glucose uptake, particularly in skeletal muscle and adipose tissues that are largely responsible for systemic glucose homeostasis (11). Inhibiting WT PI3Kα signaling causes acute insulin resistance and promotes hyperglycemia, which are key challenges with the PI3Kα inhibitor class (12). As such, updated expert recommendations indicate that alpelisib should not be prescribed to patients with poorly controlled diabetes (13), who comprise up to 31% of the overall breast cancer population (14). In clinical studies, patients with a history of diabetes were found to be at greater risk of hyperglycemia and drug discontinuation (15). Even with inclusion restrictions, real-world evidence has shown hyperglycemia in the majority of patients treated with alpelisib, as well as high rates of safety-related dose reduction and permanent discontinuations, up to 42% and 46%, respectively (15, 16). The management of alpelisib-associated metabolic dysfunction is complex, and current recommendations include frequent blood glucose measurements, a low-carbohydrate/ketogenic diet, and the use of antidiabetic medications (including insulin) either prophylactically or in treatment management (13). Another concern is that hyperinsulinemia has been shown to counteract the efficacy of PI3K inhibition in tumor models (17). Other common alpelisib-related toxicities (e.g., gastrointestinal disturbance, rash) are also thought to be directly related to the inhibition of WT PI3Kα (18, 19).

Selective targeting of mutant PI3Kα is expected to improve antitumor activity and reduce toxicity (12, 20). To test this hypothesis, we developed STX-478, a second-generation, allosteric, mutant-selective PI3Kα inhibitor that interacts with a previously undescribed allosteric pocket within PI3Kα. Preclinical results presented here show that STX-478 selectively inhibited all PI3Kα kinase-domain mutant forms *in vitro*, and potently inhibited the growth of cell lines with both helical- and kinase-domain mutations. In human tumor xenograft models harboring kinase- and helical-domain PI3Kα mutations, STX-478 demonstrated efficacy similar or superior to high-dose alpelisib while sparing metabolic dysregulation. When STX-478 was combined with fulvestrant and/or cyclin-dependent kinase (CDK) 4/6 inhibitors, robust and durable tumor regressions were achieved that persisted after the end of treatment. Our data suggest that the selective inhibition of mutant PI3Kα minimizes metabolic dysfunction and significantly improves the therapeutic index over current clinical treatment for PI3Kα-mutant cancers.

## RESULTS

### STX-478, a Mutant-Selective, Allosteric PI3Kα Inhibitor

**Figure 1.**
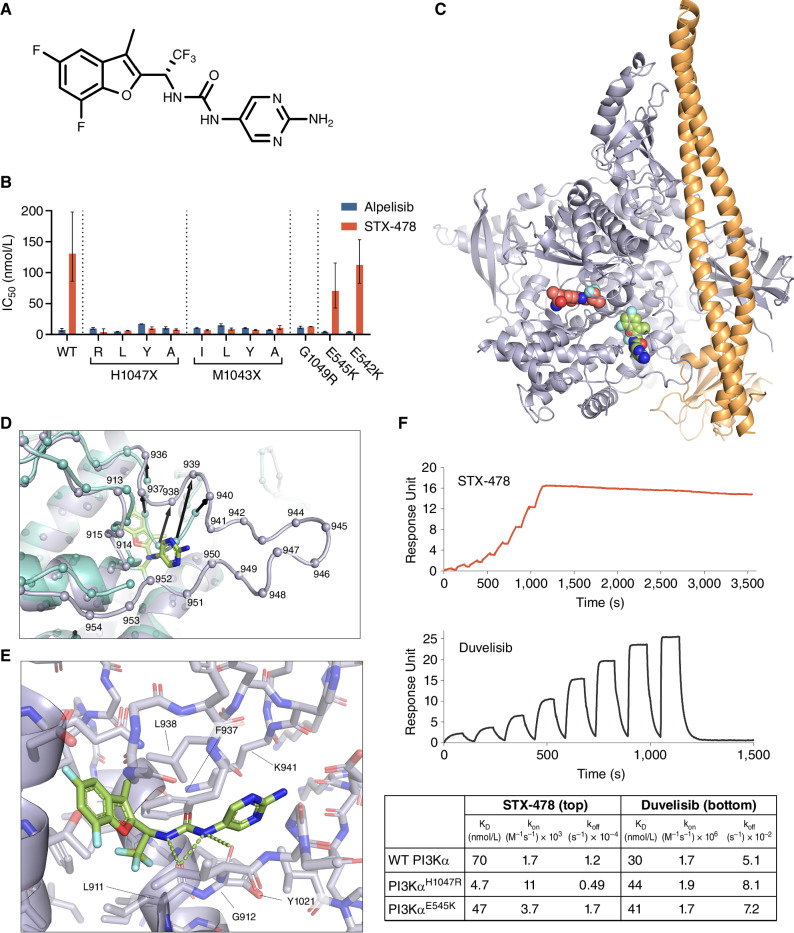
STX-478 binds a novel allosteric site on PI3Kα and achieves mutant selectivity through differential binding kinetics. **A,** The molecular structure of STX-478. **B,** Inhibition of enzyme activities of WT PI3Kα as well as kinase-domain and helical-domain mutant proteins by alpelisib and STX-478 showing the geometric mean and standard deviation. **C,** The 2.9Å X-ray structure of PI3Kα (p110 purple, p85 orange) with GDC-0077 (red spheres) bound in the ATP site and STX-478 (green spheres) bound in an allosteric site. **D,** Arrows indicate Cα (spheres) movements of ≥3Å between aligned H1047R (teal, Protein Data Bank 3HHM) and the STX-478–bound structure (purple). 3HHM lacks residues 941–952 for direct comparison. **E,** Detailed view of bound STX-478. Labeled residues significantly contribute to compound binding. **F,** SPR sensorgrams for STX-478 (top) and duvelisib (bottom) binding to PI3Kα H1047R–mutant protein. K_D_, k_on_, and k_off_ values as determined by single-cycle kinetics SPR for binding to WT, H1047R-mutant, and E545K-mutant PI3Kα proteins are shown in the table below the sensorgrams.

The initial chemical matter leading to STX-478 (Fig. 1A) was identified following a comprehensive hit-finding strategy that will be described in detail in a subsequent publication. The biochemical potency and mutant selectivity of STX-478 were evaluated in a panel of common oncogenic-mutant PI3Kα forms and compared with alpelisib as a reference (Fig. 1B). STX-478 was found to be a potent and selective inhibitor of all kinase-domain mutant PI3Kα forms found in cancer, including the most common variant H1047R (IC_50_ = 9.4 nmol/L), with 14-fold selectivity over WT PI3Kα (IC_50_ = 131 nmol/L). Under these same assay conditions, STX-478 was less potent against E542K (IC_50_ = 113 nmol/L) and E545K (IC_50_ = 71 nmol/L) helical-domain mutants, whereas alpelisib showed no mutant selectivity as previously reported (21).

STX-478 also demonstrated exquisite kinome-wide selectivity (Supplementary Fig. S1A). A biochemical screen using 373 kinases representing approximately 70% of the human kinome, including PI3Kβ, PI3Kδ, and PI3Kγ isoforms, showed that only the AurB kinase was inhibited by >50% at 10 μmol/L (IC_50_ = 1.6 μmol/L). Subsequent follow-up confirmed that STX-478 showed little AurB inhibition in cells at concentrations up to 10 μmol/L (Supplementary Fig. S1B).

X-ray cocrystals were generated to identify the binding site of STX-478 on PI3Kα. Compared with published PI3Kα structures, the cocrystal structure of H1047R with STX-478 revealed that the compound occupies a cryptic allosteric site (Fig. 1C; Supplementary Table S1) that forms due to a major conformational shift in residues 936–940, along with other smaller, local rearrangements (Fig. 1D). Specifically, residues F937 and L938 occupy positions that would directly clash with STX-478. Rearrangement of side- and main-chain atoms of these residues resulted in the repositioning of F937 and L938 to create space for the allosteric site. Additionally, relative to existing H1047R structures (3HHM and 3HIZ; ref. 22), the activation loop is better resolved in the STX-478 costructure.

STX-478 makes several specific contacts within the alloste­ric site (Fig. 1E). The benzofuran and trifluoromethyl moieties position at the deepest point of the pocket, anchoring against hydrophobic and aromatic residues. The urea present in STX-478 forms a bifurcated hydrogen bond with the L911 backbone carbonyl and a suboptimal hydrogen bond with the G912 carbonyl. This hydrogen bonding secures the inhibitor's central linker with the pyrimidine positioned closer to the protein's surface. The K941 side chain forms van der Waals contacts across the pyrimidine ring in an orientation roughly 180 degrees from the typical WT conformation of this side chain. The K941 side-chain nitrogen is positioned within hydrogen-bonding distance of the D1018 backbone carbonyl, stabilizing the protein in this conformation. A small shift in the Y1021 side chain relative to the apo conformation stabilizes the opposite side of the STX-478 pyrimidine. Comparison between WT (Supplementary Table S1) and H1047R cocrystal structures with STX-478 revealed no significant difference in the allosteric site. Kinase-domain alignment revealed no conformational variability in the domain (RMSD 0.3Å). Similarly, a detailed analysis of the allosteric site and key STX-478 interactions showed no difference between the WT- and H1047R-bound structures (Supplementary Fig. S2).

Because X-ray crystallography did not identify structural differences in STX-478 binding to mutant compared with WT PI3Kα, we asked if differences in binding kinetics could explain mutant selectivity. Surface plasmon resonance (SPR) assay results confirmed the potency and mutant selectivity observed in the biochemical assays, indicating that STX-478 has low nanomolar binding affinity (equilibrium dissociation constant, K_D_ = 4.7 nmol/L) for the H1047R mutant, 15-fold lower binding affinity for WT PI3Kα (K_D_ = 70 nmol/L), and intermediate binding to the helical-domain mutant E545K (K_D_ = 47 nmol/L). However, SPR analysis further revealed that STX-478 has faster association constants (k_on_) for H1047R and E545K compared with WT PI3Kα (Fig. 1F). In contrast, duvelisib, a nonselective, orthosteric PI3Kα inhibitor, showed approximately equal binding affinity and kinetics for mutant and WT PI3Kα. These data indicate that STX-478 mutant selectivity is derived from kinetic differences in binding between mutant and WT PI3Kα and suggest that the allosteric site occupied by STX-478 is more accessible in mutant forms of the enzyme.

### STX-478 Selectively Targets PI3Kα Activity and Cell Viability in PI3Kα-Mutant Cells

**Figure 2.**
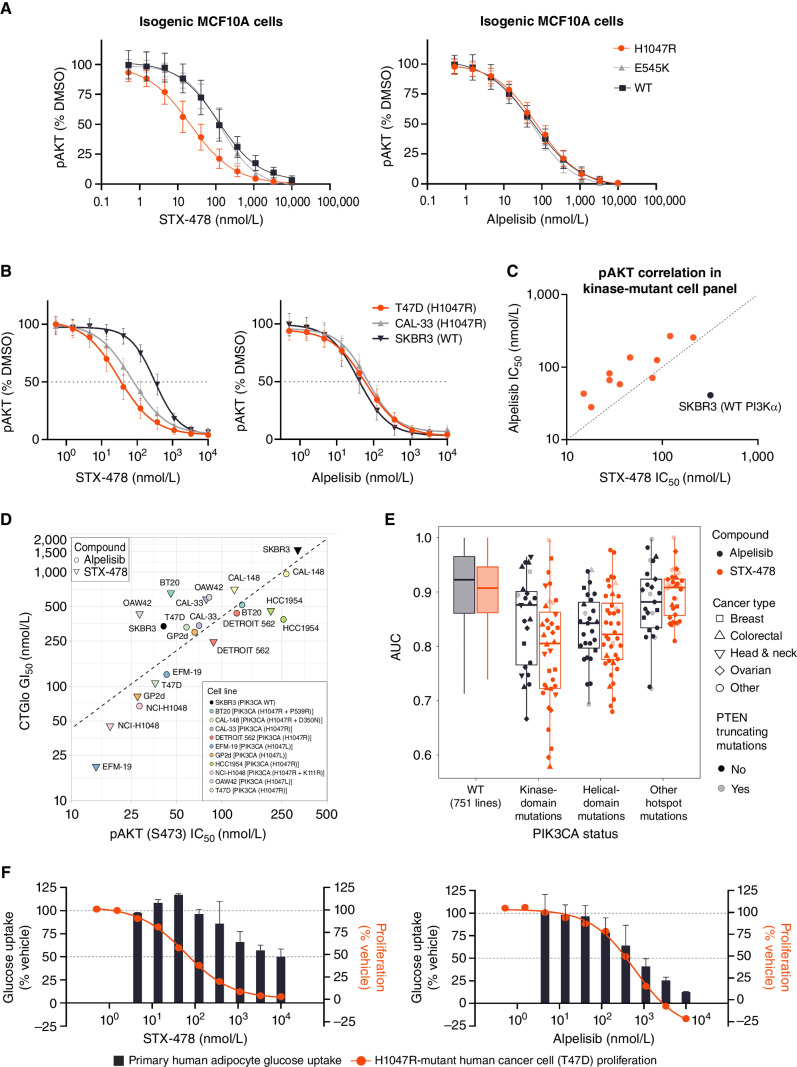
Assessment of STX-478 mutant selectivity by profiling target engagement and functional activity in cellular assays. **A,** pAKT inhibition dose–response curves (HTRF assay) in MCF10A isogenic cell lines. **B,** Representative pAKT inhibition dose–response curves (HTRF assay). **C,** Correlation plot comparing alpelisib and STX-478 potency (pAKT HTRF assay) in a panel of kinase-domain mutant cell lines (orange dots) and WT PI3Kα SKBR3 (black dots) described in Supplementary Table S1. **D,** Correlation plot of alpelisib and STX-478 comparing pAKT IC_50_ at 1 hour and viability [CellTiter-Glo (CTGlo)] GI_50_ at 72 hours across the indicated panel of cell lines. **E,** Sensitivity of cancer cell lines to STX-478 and alpelisib grouped by cancer type and *PIK3CA* and *PTEN* mutational status. Screening was performed by the Broad Institute using the PRISM platform. **F,** Two-hour glucose uptake in primary human adipocytes as indicated in the bar graph (percent vehicle response). Seventy-two-hour viability data in H1047R PI3Kα-mutant T47D cell line are overlaid in the orange dose–response curve for comparison.

STX-478 and alpelisib were studied in isogenic MCF10A cells by measuring phosphorylated (serine 473) AKT (pAKT) as a marker of target engagement with the PI3Kα/AKT pathway. Consistent with biochemical data (Fig. 1), STX-478 demonstrated selectivity for MCF10A cells harboring the H1047R kinase-domain mutation, but not the E545K helical-domain mutation, whereas alpelisib was equipotent against mutant and WT lines (Fig. 2A). Target engagement studies were extended to a panel of 10 human tumor cell lines harboring PI3Kα kinase-domain mutations (Supplementary Table S2). In addition to H1047R, the panel included three cell lines carrying the second most common kinase-domain mutation, H1047L (EFM-19, GP2d, and OAW42 cell lines), as well as three double-mutant forms (BT20, CAL-148, and NCI-H1048 cell lines). The panel also included SKBR3, breast cancer cells that depend on WT PI3Kα activity to drive oncogenic growth, as an antitarget comparator (8, 9). STX-478 effectively inhibited kinase-domain mutant-PI3Kα activity across the panel of cell lines, with IC_50_ values ranging from 15 to 319 nmol/L, which was similar to alpelisib IC_50_ values of 28 to 268 nmol/L (Supplementary Table S2). STX-478 was more potent than alpelisib in nine of the 11 cell lines, a notable exception being SKBR3, which was expected based on the reduced selectivity of STX-478 against WT (correlation plot Fig. 2B; Supplementary Table S2), and consistent with the isogenic MCF10A studies (Fig. 2A). In the ER^+^HER2^−^ breast cancer benchmark T47D (H1047R PI3Kα) cell line, STX-478 was 9-fold selective over SKBR3 (WT PI3Kα) cells (Fig. 2C). In contrast, alpelisib showed no selectivity between mutant- and WT-driven cell lines. When cell viability was studied in the same PI3Kα-dependent cell lines, there was a strong correlation between target engagement (pAKT) and cell viability [Pearson correlation coefficient = 0.8 (log scale); Fig. 2D].

STX-478 activity was next evaluated in a high-throughput cell viability panel of approximately 900 tumor cell lines to identify markers of sensitivity and draw comparisons with alpelisib. Alpelisib was previously shown to selectively inhibit the proliferation of PI3Kα-mutant cell lines (23), and our results confirmed this selectivity (Fig. 2E). STX-478 displayed a similar selectivity pattern that suggested that PI3Kα-mutant cells depend on this oncogenic driver for growth. Although our biochemical data found STX-478 to be less potent against the PI3Kα helical-domain mutant enzyme compared with the kinase-domain mutant (Figs. 1C and 2A), the potency was sufficient to result in antiproliferative activity in helical-domain mutant tumor cell lines. As previously reported, *PTEN*-inactivating mutations conferred resistance to PI3Kα inhibition in this study (*P* < 0.0081; ref. 24).

We next sought to characterize the effects of STX-478 on WT PI3Kα in a nontransformed, physiologically relevant setting by assessing insulin-mediated glucose uptake in primary human subcutaneous adipocytes (25). Adipocytes were pretreated with alpelisib or STX-478 and then supplemented with [^3^H]-2-deoxy-glucose and 10 nmol/L insulin. Alpelisib inhibited glucose uptake at concentrations as low as 100 nmol/L, with nearly complete inhibition at 10 μmol/L, whereas the concentration of STX-478 needed to attain 50% inhibition (EC_50_) was ≥10 μmol/L (Fig. 2F). The maximum effect (E_max_) of STX-478 for glucose uptake was 38%, whereas alpelisib caused deeper suppression with an E_max_ of 88%. An overlay of the cell viability dose–response curve obtained in T47D cells (Fig. 2D) illustrates the potentially improved therapeutic index of STX-478 relative to alpelisib in relevant human cell systems.

### STX-478 Treatment Yields Robust Antitumor Efficacy in PI3Kα-Mutant Tumors without Metabolic Dysregulation in Mice

Pharmacologic characterization of STX-478 *in vivo* was designed to establish the metabolic safety and antitumor efficacy profile of STX-478 compared with alpelisib. A 50 mg/kg once-daily (q.d.) dose of alpelisib was chosen, as this dose was efficacious in published mouse xenograft models, albeit accompanied by glucose dysregulation (21). At this dose, the alpelisib plasma exposure (AUC) in mice (∼75,000 ng*h/mL) exceeded the exposure of the maximum approved human dose by approximately 2-fold (∼33,000 ng*h/mL; ref. 26); therefore, a lower dose (20 mg/kg) was included to approximate the clinically relevant exposure. Based on the STX-478 pharmacokinetic (PK) profile, 30- and 100-mg/kg q.d. doses were expected to bracket the 80% inhibitory concentration (IC_80_) of relevant cancer cell lines, whereas the 300 mg/kg q.d. dose exceeded this (Supplementary Fig. S3).

**Figure 3.**
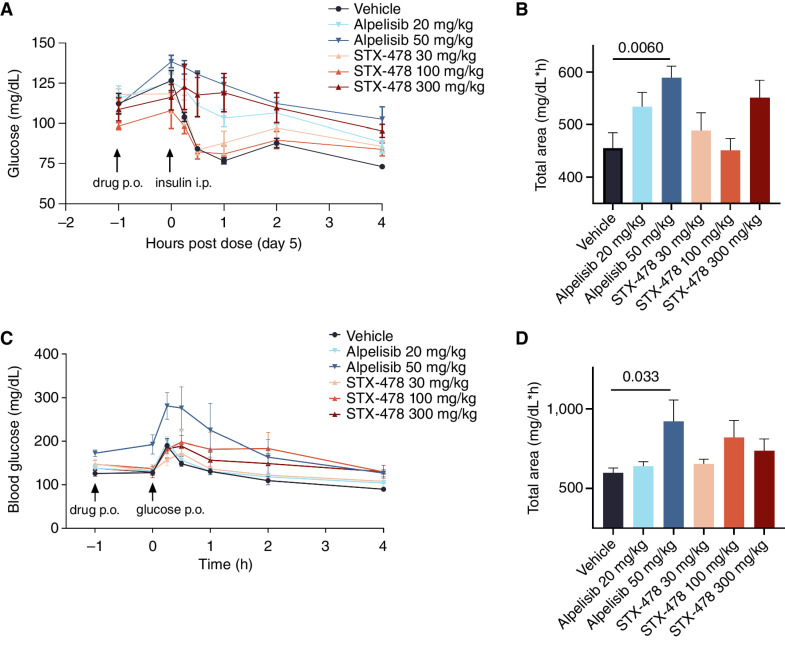
The effect of STX-478 and alpelisib on glucose homeostasis. **A,** Tumor-naive female BALB/c nude mice were dosed for 5 days as indicated (*n* = 5/group) and subjected to an ITT. On day 5, animals were fasted for 6 hours and dosed with drug by oral administration (p.o.) as indicated 1 hour prior to the end of the fast (−1 hour). At *T* = 0, animals were dosed with 0.75 U/kg by intraperitoneal (i.p.) insulin administration. Blood glucose levels were monitored over time, with group mean and standard error mean (SEM) indicated. **B,** AUCs were calculated from **A**; the AUC of each treatment group was compared with the vehicle group using ordinary one-way ANOVA and Dunnett multiple comparisons tests. **C,** An OGTT was performed similarly to the ITT (**A**), except at *T* = 0, when mice were dosed with 2 g/kg glucose and blood glucose was monitored over time, with group mean and SEM indicated. **D,** AUCs were calculated from **C** and analyzed by one-way ANOVA as described in **B**.

With regard to metabolic control, the primary consequence of WT PI3Kα inhibition is to block insulin action (e.g., insulin resistance), impairing glucose disposal and causing hyperglycemia (2, 11). The impact of alpelisib and STX-478 doses on insulin sensitivity was analyzed using an insulin tolerance test (ITT) and oral glucose tolerance test (OGTT) following 5 days of repeat dosing in non–tumor-bearing, female BALB/c nude mice, the sex/strain frequently used in our xenograft studies. Alpelisib treatment resulted in a dose-dependent reduction in glucose disposal in both the ITT and OGTT, consistent with insulin resistance (Fig. 3A–D, respectively). In contrast, STX-478 treatment was not associated with significant changes in glucose AUC, although there was a nonstatistically significant increase at the 300 mg/kg dose in the ITT. Notably, STX-478 had no effect on body weight or fasting plasma glucose after 5 days of treatment. These studies established that repeat doses of STX-478 at 100 mg/kg q.d. were well tolerated without metabolic dysregulation, whereas alpelisib caused overt insulin resistance at 50 mg/kg dose levels. We confirmed that STX-478 has similar, or even higher potency, against mouse PI3Kα (IC_50_ mouse = 87 nmol/L vs. human = 139 nmol/L), confirming that mouse WT PI3Kα is a good surrogate for humans and that the therapeutic index can be reasonably evaluated using human tumor xenografts in mice.

### Benchmarking STX-478 in the CAL-33 (H1047R PI3Kα) Human HNSCC Xenograft Model

The CAL-33 (H1047R PI3Kα) HNSCC cell model displayed intermediate sensitivity to STX-478 in culture (Fig. 2D) and was selected to benchmark therapeutic activity and pharmacodynamic (PD) biomarkers. The study consisted of three arms: an efficacy group in which animals received test articles for 28 days; a PK/PD group in which animals received STX-478 or alpelisib treatment for 3 days; and a group in which tumor-bearing animals received an oral bolus of [U-^13^C]-glucose to assess the effects of alpelisib and STX-478 on glucose uptake and glucose oxidation in target tissues.

**Figure 4.**
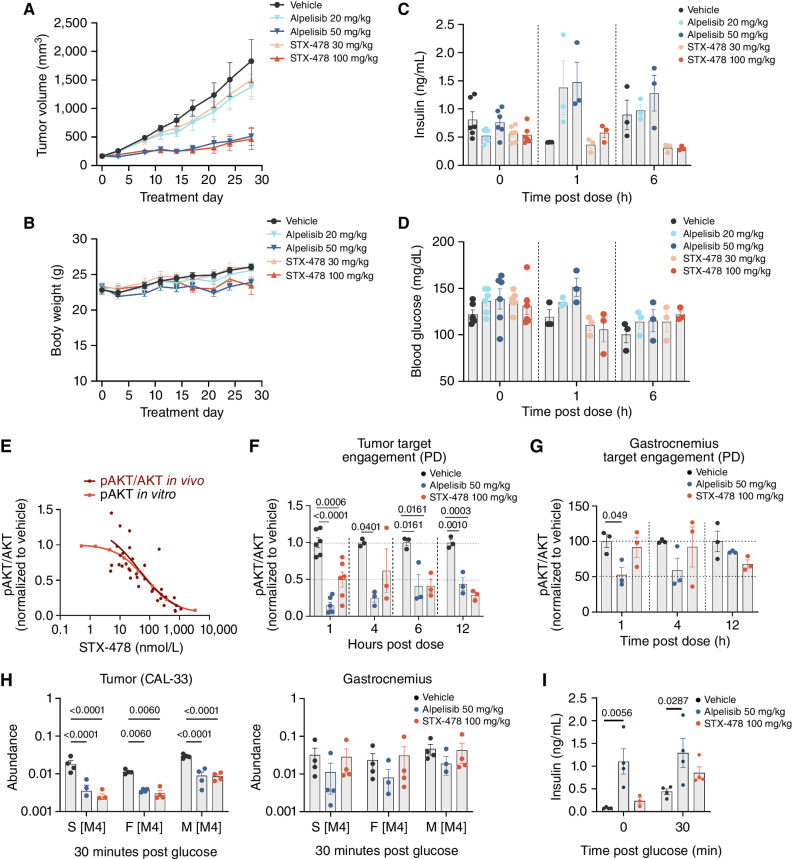
Efficacy and PK/PD profiling of STX-478 and alpelisib in CAL-33 xenograft tumors. **A** and **B,** CAL-33 xenograft tumors were established in female BALB/c nude mice. Animals were randomized into treatment groups (*n* = 6) at approximately 160 mm^3^ and treated as indicated. Tumor (**A**) and body weight (BW; **B**) were measured 2×/week with group mean and standard error mean (SEM) shown. **C** and **D,** Mice bearing CAL-33 tumors were treated with oral (p.o) daily dosing of indicated treatment for 3 days, and serum insulin (**C**) and blood glucose (**D**) were measured, with individual values, group mean, and SEM shown with each treatment group compared with the vehicle group using ordinary one-way ANOVA and Dunnett multiple comparisons tests. **E,** pAKT (S473) was measured by Western blot in all CAL-33 tumors from mice in the 28-day efficacy study (30 and 100 mg/kg dose groups) and 3-day PK/PD study (30, 100, and 300 mg/kg dose groups). pAKT (S473) and total AKT were measured in CAL-33 tumors by Western blot. The normalized pAKT (S473) levels were plotted against the unbound concentration (red). For reference, the *in vitro* pAKT dose response in CAL-33 cells (orange curve) has been added (as in Fig. 2). **F,** pAKT levels in CAL-33 tumors showing only 100 mg/kg STX-478 and 50 mg/kg alpelisib over time. **G,** pAKT (S473) was measured by Western blot from the gastrocnemius of mice represented in **E** and **F**. Significance was measured using ordinary one-way ANOVA and Dunnett multiple comparisons tests. **H,***N* = 4 mice were fasted for 4 hours and then administered vehicle, alpelisib (50 mg/kg), or STX-478 (100 mg/kg), followed 1 hour later with p.o. administration of [U-^13^C]-glucose. After 30 minutes, the tissues were collected and analyzed by mass spectrometry. The abundance of labeled [M+4] succinic acid (S), fumaric acid (F), and malic acid (M) in tumor and gastrocnemius is shown. Individual values, group mean, and standard error mean are shown with each treatment group compared with the vehicle group using two-way ANOVA and Holm–Šídák posttest. **I,** Plasma insulin was measured immediately before and 30 minutes after labeled-glucose administration.

In the efficacy arm, there was a dose-dependent reduction in tumor volume with both compounds: STX-478 30 mg/kg showed similar efficacy to alpelisib 20 mg/kg, and STX-478 100 mg/kg showed efficacy similar to alpelisib 50 mg/kg (Fig. 4A). All treatments were well tolerated, with no change in body weight (Fig. 4B). Alpelisib raised serum insulin at 1 hour after dose on day 28 (*P* = 0.0585; Fig. 4C), with a similar trend in glucose (*P* < 0.083; Fig. 4D).

PD biomarkers of target engagement (pAKT/AKT ratio) and STX-478 plasma and tumor drug levels were measured 1, 4, and 12 hours after dose on day 3 (PK/PD group), and 1 and 6 hours after final dose (day 28). The curve–fit relationship between tumor drug concentration and pAKT/AKT levels in CAL-33 tumor xenografts and *in vitro* is shown in Fig. 4E. The calculated IC_50_ from this curve fit was 45 nmol/L in tumor compared with 18 nmol/L from cell culture when corrected for matrix binding (Pearson *r* correlation coefficient = −0.613; *P* < 0.0001), demonstrating a convincing *in vitro*–*in vivo* correlation. In this xenograft study, tumor growth inhibition (TGI) of 82% in the STX-478 100 mg/kg q.d. dose group was associated with average pAKT suppression of 57% (treated/vehicle AUC_1–12h_); for the alpelisib 50 mg/kg q.d. group, a TGI of 79% was associated with average pAKT suppression of 66% (Fig. 4F). Unlike alpelisib, STX-478 did not reduce skeletal muscle pAKT/AKT (Fig. 4G).

Inhibition of PI3Kα is known to suppress glucose metabolism in tumor and host tissues (17, 26–28). To assess the effects of alpelisib and STX-478 on this process, CAL-33 tumor–bearing mice were pretreated with drug or vehicle and then given [U-^13^C]-glucose by oral gavage. After 30 minutes, the tumor and skeletal muscle metabolites were extracted and quantified using liquid chromatography–mass spectrometry (29). The oral bolus led to robust labeling of over 70% of circulating glucose carbon in all groups (Supplementary Fig. S4). In tumors, both alpelisib and STX-478 significantly reduced the incorporation of [^13^C]- into tricarboxylic acid intermediates, a marker of glucose oxidation (Fig. 4H). However, only alpelisib-treated mice demonstrated reductions in glucose oxidation in the skeletal muscle (Fig. 4H). This reduction occurred despite higher levels of circulating insulin in this group (30 minutes alpelisib vs. vehicle), suggesting insulin resistance in this tissue (Fig. 4I). These data indicate STX-478 selectively inhibits mutant PI3Kα but not the WT enzyme found in host tissues.

### STX-478 Is Efficacious across a Panel of PI3Kα-Mutant Cell-Derived Xenograft and Patient-Derived Xenograft Tumors without Evidence of Insulin Resistance

**Figure 5.**
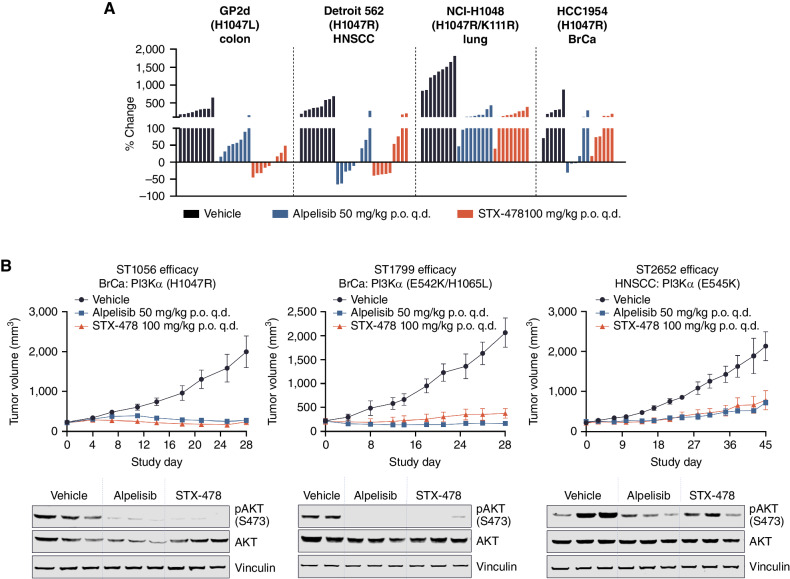
The efficacy of STX-478 is similar or superior to high-dose alpelisib across PI3Kα-mutant tumor xenografts while sparing insulin resistance (p.o., oral administration; q.d., daily). **A,** Final tumor volume percent change is represented for GP2d, Detroit 562, NCI-H1048, and HCC1954 CDX tumors. BrCa, breast cancer. **B,** Tumor volumes over time from PDX models (*N* = 3/group) harboring PI3Kα mutations in the kinase domain (ST1056: H1047R PI3Kα), kinase and helical domains (ST1799: E542K/H1065L PI3Kα), and helical domain (ST2652: E545K PI3Kα) treated with either vehicle, STX-478, or alpelisib. Statistical significance was calculated using two-way ANOVA and Dunnett multiple comparisons tests. End-of-study tumors were harvested 4 hours after the final dose, and pAKT (S473) was analyzed by Western blot. IsoSeq analysis of a tumor from model ST1799 showed that the E542K/H1065L PI3Kα mutations are in *cis*. AKT and pAKT (S473) were blotted separately, and a representative vinculin blot is shown.

This detailed metabolic characterization of STX-478 and activity in the CAL-33 xenograft model established that the optimal STX-478 dose was 100 mg/kg q.d. and demonstrated that STX-478 was more efficacious than a clinically matched dose of alpelisib (20 mg/kg). Therefore, the STX-478 100 mg/kg q.d. dose was carried forward into a panel of PI3Kα-mutant cell-derived xenograft (CDX) and patient-derived xenograft (PDX) models representing colon cancer (GP2d), lung cancer (NCI-H1048), HNSCC (Detroit 562), and HR^−^HER2^+^ breast cancer (HCC1954). The efficacious high-dose alpelisib (50 mg/kg q.d.) was included as a benchmark, and all studies were conducted in BALB/c nude mice. The key efficacy endpoint was TGI or regression, and tolerability measures included body weight and insulin levels at 1 hour after dose at the end of the study. No treatment-specific effects on body weight were noted with either STX-478 or alpelisib across studies (Supplementary Fig. S5). The TGI of STX-478 100 mg/kg q.d. treatment was similar or superior to 50 mg/kg q.d. alpelisib treatment (Fig. 5A; Supplementary Table S3; and Supplementary Fig. S5A–S5D). STX-478 demonstrated robust efficacy in GP2d tumor xenografts that express H1047 L PI3Kα, the second-most prevalent kinase-domain mutation. The waterfall plot shows tumor regressions in half of the STX-478–treated animals, whereas no regression was seen with alpelisib treatment (Fig. 5A). In the Detroit 562 HNSCC model, compelling efficacy was seen with both compounds, including tumor regressions in five of the nine study animals. Similarly, STX-478 and alpelisib treatment provided comparable tumor growth control in NCI-H1048 lung carcinoma, which contains a double (H1047R/K411R) PI3Kα mutation, as well as in the HCC1954 HR^+^HER2^+^ breast cancer model (Fig. 5A). In every study described above, significant increases in serum insulin were observed in animals dosed with alpelisib 50 mg/kg 1 hour after dose, whereas STX-478 100 mg/kg was not associated with elevated insulin (Supplementary Table S3). Although both alpelisib and STX-478 treatment caused significant TGI, HCC1954 cells showed the lowest response of all cell lines tested in the CDX panel. HCC1954 cells were also less sensitive to both agents in cell culture (Fig. 2C) and may reflect reduced efficacy of PI3Kα inhibitor monotherapy in HER2^+^ cancers due to compensatory HER3 activation (30, 31).

STX-478 and alpelisib monotherapies were evaluated in two breast cancer PDX models, ST1056 [kinase-domain mutant (H1047R)] and ST1799 [a double mutant with a hotspot helical-domain mutation and a kinase mutation of unknown significance (E542K/H1065L)]. We also evaluated a third HNSCC xenograft model (ST2652) carrying a helical-domain mutation (E545K). STX-478 was highly efficacious in all three models, including the helical-domain mutant tumors, with efficacy similar to alpelisib (Fig. 5B) and no adverse effect on body weight (Supplementary Fig. S5E). For both STX-478 and alpelisib, a reduction in pAKT/AKT was seen 4 hours after dose (Fig. 5B). Given the importance of this finding, we examined a third helical-domain mutant PDX cell line (ST986, E542K), which is a HER2^+^ cancer. STX-478 (100 mg/kg) and alpelisib (50 mg/kg) demonstrated similar TGI (Supplementary Fig. S5F), providing a compelling rationale to test STX-478 in patients with either kinase or helical-domain mutant tumors.

### Safety and Efficacy of Clinically Relevant Combination Therapies

Although alpelisib monotherapy yielded tumor responses in heavily pretreated ER^+^HER2^−^ advanced breast cancer in phase I (26) and phase II (32) studies, the greatest therapeutic response tested was observed when alpelisib was administered in combination with fulvestrant (33). Preclinical studies further suggested that a CDK4/6 inhibitor could also be an efficacious combination partner with a PI3Kα inhibitor (34). To gauge the efficacy potential of combinations, we studied STX-478 and fulvestrant monotherapy and combination therapy in the T47D cell xenograft model. In the ER^+^HER2^−^ PDX model, STX-478, fulvestrant, and palbociclib monotherapies, as well as their pairwise and triple combinations, were assessed.

**Figure 6.**
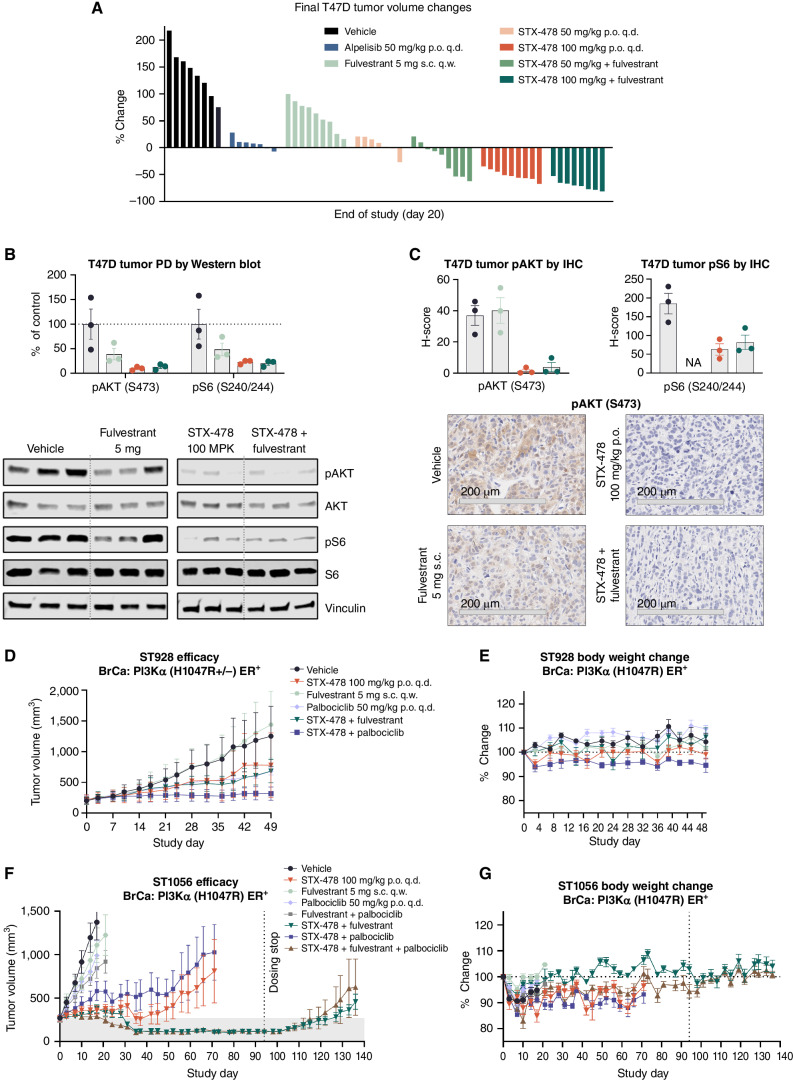
STX-478 combination therapies in ER^+^ breast cancer models with fulvestrant and palbociclib. **A,** T47D xenograft tumors were established in female NOD scid gamma immunodeficient (NSG) mice. When tumors reached approximately 200 mm^3^, mice were randomized and treated as indicated (p.o., oral administration; q.d., daily; q.w., weekly; s.c., subcutaneous injection). Data represent the percent change in tumor volume of individual tumors on day 20 relative to randomization. **B** and **C,** NSG mice bearing T47D tumors were given a single dose of vehicle or compound(s) as indicated. Tumors were harvested 4 hours after STX-478 and 24 hours after fulvestrant for Western blot of pAKT (S473) and S6 (S240/244; **B**), and IHC of pAKT (S473; **C**). AKT and S6, and pS6 and pAKT (S473) groups were blotted and analyzed together. All samples for a given protein were run on the same gel. A representative vinculin blot is shown. For clarity, images were cropped to remove other compounds. Representative 20× IHC images are shown with a 200-μm scale bar. **D,** ST-928 xenograft tumors were established in BALB/c nude mice and randomized to indicated treatment groups when tumors reached ∼210 mm^3^. BrCa, breast cancer. **E,** Percent change in body weight for **D**. **F,** ST1056 xenograft tumors were established in BALB/c nude mice. Mice were randomized to treatment groups as indicated when tumors reached approximately 260 mm^3^. Mice were treated for 94 days or until removal from the study due to tumor volume. After 94 days, treatment was ended, and tumors were monitored for regrowth. **G,** Percent change in body weight for **F**.

The T47D cell line represents an important estrogen-dependent, ER^+^HER2^−^ breast cancer benchmark to test the mutant PI3Kα mechanism and previously characterized with alpelisib treatment (21). STX-478 monotherapy demonstrated dose-dependent TGI, with the 50 mg/kg q.d. dose achieving TGI similar to high-dose alpelisib (50 mg/kg q.d.) and the STX-478 100 mg/kg q.d. dose yielding significant tumor regression in every animal (Fig. 6A). Fulvestrant monotherapy provided only ∼50% TGI, while adding 50 or 100 mg/kg dose levels of STX-478 to fulvestrant treatment led to 20% and 70% regressions, respectively. Fulvestrant monotherapy and STX-478 combinations were well tolerated (Supplementary Fig. S6). Single-dose PK/PD measures were performed in tumors collected 4 hours after administration of STX-478 100 mg/kg and 24 hours after fulvestrant. Biomarkers of PI3Kα pathway activity, pAKT and pS6, were modestly reduced by fulvestrant, whereas STX-478 led to significant inhibition as assessed by Western blot and IHC (Fig. 6B and C, respectively).

Combination studies were extended to breast cancer PDX models, which may be a more predictive translational model for oncology drug development (35). In ST928, an H1047R, ER^+^HER2^+^ tumor, STX-478 and palbociclib monotherapy each provided modest TGI, but near-complete TGI was observed in combination (Fig. 6D). Fulvestrant provided no monotherapy or combination benefit, but all combinations were well tolerated (Fig. 6D and E). We also tested STX-478 combinations in an aggressive ER^+^HER2^−^ breast cancer, ST1056. Tumors grew rapidly in the vehicle, palbociclib, and fulvestrant monotherapy groups, as well as the fulvestrant and palbociclib combination group, requiring animals to be removed from the study by day 17 (Fig. 6F). STX-478 monotherapy led to durable tumor growth suppression for 49 days or more. The addition of palbociclib to STX-478 treatment provided no additional benefit in any group. The most remarkable responses occurred with the combination of STX-478 and fulvestrant. Tumor suppression continued to day 94 in every animal until treatment was stopped, and modest regrowth was observed after only a month off treatment when the study ended (Fig 6F). All treatments, including the triple combination of STX-478, fulvestrant, and palbociclib, were well tolerated for over >90 days of dosing (Fig. 6G).

## DISCUSSION

The development of PI3Kα inhibitors to treat cancer has been a journey of incremental improvements. First-generation orthosteric inhibitors such as buparlisib lacked isoform selectivity and were associated with an unacceptable toxicity profile despite having promising signs of efficacy (36, 37). The development of alpelisib with α-isoform selectivity led to an effective treatment option for patients with PI3Kα-mutant, ER^+^HER2^−^ breast cancer. However, inhibition of the WT PI3Kα in metabolic tissues restricts the full potential of this therapy. STX-478 provides the next logical step in the evolution of PI3Kα inhibitors and allows us to now test the hypothesis that a mutant-selective PI3Kα inhibitor will significantly improve the therapeutic window and transform the treatment paradigm for PI3Kα-mutant cancers.

Here we show the first crystal structure of PI3Kα bound to STX-478 within a novel, cryptic allosteric site. This site is present in both mutant and WT enzyme forms, and it is distinct from the previously described PIK108 allosteric site [Protein Data Bank (PDB) identifier 4A55; ref. 38]. STX-478 binding kinetics revealed a faster association rate with the H1047R mutant and a slightly faster association rate with the E545K mutant, suggesting that these mutations open the allosteric site more readily, enabling the mutant selectivity of STX-478. This site has remarkable plasticity, allowing STX-478 to sit adjacent to the activation loop after rearrangement of several residues. Cellular pharmacology assay results showed STX-478 to have equivalent or superior target engagement as alpelisib in cell lines harboring kinase-domain mutants of PI3Kα, and this correlated with cell viability.

In a large-scale screen comprising over 900 tumor cell lines, STX-478 selectively inhibited the proliferation of cell lines with kinase-domain and helical-domain mutations compared with cells expressing WT PI3Kα. This pattern is similar to results achieved with alpelisib and consistent with clinical data showing that efficacy is superior in tumors expressing PI3Kα mutations (23). The sensitivity of helical-domain mutant cell lines to STX-478 was somewhat unexpected given the decreased potency relative to kinase-domain mutations in biochemical assays and the isogenic cell setting. Nonetheless, it is clear from the PDX studies that STX-478 has significant antitumor activity in both kinase- and helical-domain mutant xenografts at a dose that does not cause metabolic dysregulation.

Selective targeting of mutant PI3Kα with STX-478 avoided systemic metabolic dysfunction that was caused by equally efficacious doses of alpelisib. STX-478 preserved glucose uptake in human adipocytes and did not induce insulin resistance *in vivo*. The selective targeting of mutant PI3Kα was further shown by differential effects of STX-478 on pAKT/AKT suppression in tumor and muscle, and by assessing glucose oxidation in these tissues with an isotope-labeled glucose OGTT. These data indicate a superior metabolic safety profile of STX-478 relative to alpelisib, which may further enhance efficacy by blunting counterregulatory insulin spikes that diminish effectiveness in preclinical studies (17).

STX-478 monotherapy was studied in a panel of 10 CDX and PDX tumors comprising primarily breast and HNSCC tumors, with one example each for colon and lung cancers, representing prevalent PI3Kα-mutated cancer types for which there is a high unmet need. STX-478 100 mg/kg q.d. demonstrated robust efficacy in xenograft models that was similar or superior to high-dose alpelisib, giving a mouse alpelisib exposure that exceeded patient exposure by approximately 2-fold (based on AUC_24h_ with 50 mg dose). STX-478 was equally efficacious in xenografts bearing the H1047L variant, the second-most prevalent kinase-domain mutation after H1047R. Importantly, STX-478 treatment was highly efficacious in PDX tumors harboring E545K and E542K mutations, as well as in tumors containing secondary PI3Kα mutations, which occur in about 10% of primary tumors (39).

CDK4/6 inhibitors and antiestrogen therapies are important standard-of-care treatments for ER^+^ breast cancer. In the benchmark T47D ER^+^HER2^−^ breast cancer CDX model, fulvestrant monotherapy provided a moderate level of tumor growth control, whereas low-dose STX-478 monotherapy and high-dose alpelisib resulted in tumor stasis. The combination of fulvestrant with low-dose STX-478 was superior to low-dose STX-478 monotherapy, with regressions in most animals. The higher dose of STX-478 resulted in deep tumor regressions with or without fulvestrant combination therapy. Palbociclib, fulvestrant, and STX-478 monotherapies, as well as all combinations, including triple therapy, were studied in an aggressive breast cancer PDX model (ST1056). Although STX-478 monotherapy provided robust and durable responses in this model, the combination of fulvestrant and STX-478 provided exceptional tumor growth control, with regressions that were sustained in every animal for over 90 days of treatment and maintained for weeks after treatment was stopped. Although palbociclib demonstrated limited efficacy as a monotherapy or in combination with STX-478 in this PDX model, triple- combination therapy with STX-478 and fulvestrant was well tolerated in mice for over 90 treatment days. In a CDK4/6 inhibitor–sensitive PDX model, there was a combinatorial benefit between STX-478 and palbociclib with no overt toxicity. In contrast, the triple combination of alpelisib with ribociclib and fulvestrant was not tolerated in the clinic and resulted in elevated hepatobiliary toxicity, as well as increased incidence and severity of rash (40). These results highlight the challenge in developing effective combination regimens with drugs with a narrow therapeutic index and support the evaluation of STX-478 as a potentially improved combination partner in this setting. It should be noted that inavolisib, another orthosteric PI3Kα inhibitor, is currently being evaluated in a phase III clinical trial in combination with palbociclib and fulvestrant (NCT04191499), and the tolerability of this combination is yet to be defined.

STX-478 is one of four allosteric, mutant-selective PI3Kα inhibitors to recently enter clinical development, albeit neither the specific chemical entities of the three other investigational compounds nor the description of how they structurally interact with PI3Kα has been disclosed, preventing direct comparison with STX-478. A recent review summarizes data that have been disclosed at scientific conferences (20) and enables some general comparisons. STX-478 and RLY-2608 show similar binding kinetics to mutant enzymes and have inhibitory activity against both helical- and kinase-domain mutants in culture and xenografts, suggesting they could bind to the same site. On the other hand, LOXO-783 appears to have exclusive activity against the H1047R-mutated PI3Kα. In addition, STX-478 and LOXO-783 were both reported to have brain exposure, potentially making these compounds suitable to treat the significant population of patients with advanced breast cancer who develop brain metastases (20, 41). There is considerable excitement for the potential for these second-generation, mutant-selective inhibitors to bring transformational benefits to patients with PI3Kα-mutant cancers.

In conclusion, PI3Kα is a dynamic enzyme that functions in a complex lipid membrane environment, integrating inputs from numerous signaling pathways. Previous studies established that helical- and kinase-domain classes of mutations activate PI3Kα by distinct mechanisms and are differentially activated in cells (42, 43). We are just beginning to elucidate how STX-478 modulates mutant-selective PI3Kα inhibition through the cryptic pocket. Although X-ray cocrystal structures did not provide a structural explanation for mutant selectivity, differences in binding kinetics provide a strong hypothesis for kinase-domain mutant selectivity that is somewhat weaker for helical-domain mutant selectivity. Likewise, the biochemical assays and isogenic *in vitro* cell systems may be imperfect surrogates for predicting WT PI3Kα inhibition *in vivo* and the observed therapeutic index. Regardless, some *in vitro* measures clearly demonstrate a lack of potent WT inhibitory activity of STX-478, as demonstrated in insulin-mediated glucose uptake in primary human adipocytes and in the Broad Institute's PRISM cell viability panel screen, which can be viewed as a population-based model of cancer therapeutic response in which STX-478 demonstrated selective inhibition of kinase- and helical-domain mutant PI3Kα tumor cell lines compared with WT. Thus, our results are consistent with STX-478 having broad activity across kinase- and helical-domain PI3Kα-mutant tumors, with a range of activity in individual cancers not unlike alpelisib, while sparing metabolic dysfunction. STX-478 and the identification of the PI3Kα cryptic allosteric site expand our understanding of novel pharmacology, which may be leveraged to improve the treatment of cancer as well as PI3Kα-related overgrowth syndrome diseases (44).

## METHODS

### STX-478 Synthesis and Alpelisib

STX-478 was prepared according to the procedure reported in WO2022265993 (Compound 80; ref. 45). Alpelisib (>99% purity) was purchased from MedChemExpress (#HY-15244).

### X-Ray Crystallography

#### Protein Expression and Purification.

PI3Kα (1–1068; H1047R) and HIS6-tagged p85α (308–593) for crystallization were coexpressed in Sf21 insect cells in media with 1 μmol/L of GDC-0077 (inavolisib). The cells were harvested after 66 hours at 27°C and stored at −80°C until lysed in a Microfluidizer LM10 with buffer containing 50 mmol/L Na_3_PO_4_ pH 8.0, 300 mmol/L NaCl, 5% glycerol, 10 mmol/L imidazole, 2 mmol/L Tris (2-carboxyethyl) phosphine hydrochloride (TPED), 1 mmol/L MgCl_2_, 0.1% NP-40, a cOmplete EDTA-free protease inhibitor tablet (1 tablet/100 mL buffer; Roche), and 10 μmol/L GDC-0077.

The H1047R heterodimer was purified by Ni-NTA chromatography column equilibrated with 20 mmol/L Na_3_PO_4_ pH 8.0, 300 mmol/L NaCl, 5% glycerol, 20 mmol/L imidazole, and 1 mmol/L TCEP. After washing, the target protein was eluted in a buffer supplemented with 300 mmol/L imidazole. Anion exchange was performed with a HiTrap Q FF column (Cytiva) using 20 mmol/L Tris/HCl pH 8.5, 50 mmol/L NaCl, 5% glycerol, and 1 mmol/L TCEP (buffer A), to which 1 M NaCl was added for gradient elution (buffer B). Size-exclusion chromatography was used to polish and buffer exchange into 20 mmol/L Tris/HCl pH 8.0, 150 mmol/L NaCl, and 1 mmol/L TCEP. The protein complex was concentrated to 20.5 mg/mL using an Amicon Ultra concentrator and stored at −80°C until crystallization.

The WT PI3Kα (1–1068) and HIS6-tagged p85α (308–593) complex for crystallization was similarly coexpressed and purified. Proteins were expressed in HF insect cells in the presence of 50 nmol/L GDC-0077. Lysis buffer contained 50 mmol/L Na_3_PO_4_ pH 8.0, 400 mmol/L NaCl, 5% glycerol, 10 mmol/L imidazole, 1% Triton X-100, 5 mmol/L β-mercaptoethanol, 1 mmol/L Na_3_VO_4_, and cOmplete EDTA-free protease inhibitor. The heterodimer was purified with Ni-NTA affi­nity capture, followed by anion exchange (50–500 mmol/L NaCl gradient). SEC polishing was performed in 50 mmol/L HEPES pH 8.0, 150 mmol/L NaCl, and 1 mmol/L DTT.

Full-length constructs (PI3Kα 1–1068 and p85 1–724) for WT, M1043X, H1047X, and G1049R proteins were similarly coexpressed and purified with minor differences. The TEV-cleavable His tag was fused to the p110 subunit instead of p85. Full-length complexes were expressed in HF insect cells in the presence of 50 nmol/L GDC-0077. Lysis buffer contained 50 mmol/L Na_3_PO_4_ pH 8.0, 400 mmol/L NaCl, 5% glycerol, 10 mmol/L imidazole, 1% Triton X-100, 5 mmol/L β-mercaptoethanol, 1 mmol/L Na_3_VO_4_, and cOmplete EDTA-free protease inhibitor (Roche). Full-length complexes were similarly purified by Ni-NTA equilibrated in lysis buffer, washed with lysis buffer supplemented with 20 mmol/L imidazole, and eluted in 250 mmol/L imidazole. Complexes were subsequently purified by anion exchange with a HiTrap Q FF column (Cytiva) using 50 mmol/L Na_3_PO_4_ pH 8.0 with 1 mmol/L dithiothreitol (DTT; buffer A) and gradient 50 mmol/L HEPES pH 8.0, with a 150 to 500 mmol/L NaCl gradient (buffer B). Protein complexes were dialyzed into 50 mmol/L HEPES pH 8.0, 150 mmol/L NaCl, and 1 mmol/L DTT for SEC polishing.

#### Crystallization, Data Collection, and Processing.

Crystals of the PI3Kα/p85α heterodimer in complex with GDC-0077 and STX-478 were obtained at a concentration of 10 mg/mL. The heterodimer was preincubated with 0.09 mmol/L each of GDC-0077 and STX-478 (H1047R) for 1 hour or 0.1 mmol/L STX-478 for 2 hours at 4°C (WT). The H1047R sample was mixed 1:1 with reservoir solution [0.1 M MES pH 6.8, 0.5 M NaCl, and 5% (w/v) polyethylene glycol 3350] and equilibrated at 293 K over 60 μL of reservoir solution. The WT sample was mixed 1:2 with reservoir solution [0.1 M MES pH 6.8, 0.5 M sodium chloride, and 8% (w/v) PEG 3350]. Crystals were cryoprotected in a reservoir solution supplemented with 30% ethylene glycol and flash frozen in liquid nitrogen. A complete 2.9 Å dataset of a PI3Kα (H1047R)/p85α/GDC-0077/STX-478 crystal was collected at the European Synchrotron Radiation Facility (ESRF, beamline ID30A1). A 3.11 Å dataset of PI3Kα/p85α/GDC-0077/STX-478 crystal was collected at Spring8 (beamline BL45XU). Data were integrated, analyzed, and scaled by the programs XDS (46), POINTLESS, AIMLESS (47), and STARANISO (H1047R dataset only) in autoPROC (48).

#### Structure Solution and Refinement.

H1047R and WT datasets used an isomorphous reference model of PI3Kα in complex with p85α as a starting model for restrained refinement. Several rounds of refinement with the programs BUSTER RRID:SCR_015653 (H1047R) and REFMAC5 RRID:SCR014225 (WT), and modeling in COOT (RRID:SCR_01422, resulted in the final model. Atomic displacement factors were modeled with a single isotropic B-factor per atom and a single TLS group per chain (H1047R). NCS restraints were used (H1047R and WT). The final model consisted of two heterodimers in the asymmetric unit, both bound with STX-478 and GDC-0077. The chain A and B heterodimer was better resolved than the other heterodimer and was therefore used in the analysis of STX-478 binding. Statistics for the crystal structures are reported in Supplementary Table S1. Images were generated using PyMOL RRID:SCR_000305.

### SPR

Biotinylated proteins were immobilized onto a streptavidin sensor chip. Compound binding kinetics and affinities were measured in the single-cycle kinetics mode. The running buffer contained 50 mmol/L Tris pH 7.5, 150 mmol/L NaCl, 0.01% Brij 35, 1 mmol/L DTT, 1 mmol/L MgCl_2_, 0.05% Tween-20, and 2% DMSO. The assay temperature was maintained at 25^°^C, and data were fit into the 1:1 binding model.

### PI3Kα ATPase Assay

Full-length WT, M1043X, H1047X, G1049R, E545K, or E542K enzyme (1–10 nmol/L) was incubated with vehicle or compound at room temperature for 1 hour, followed by the addition of ATP (90 μmol/L final) to initiate the enzyme reaction. Assay buffer contained 50 mmol/L Tris, 150 mmol/L NaCl, 0.01% Brij 35, 15 mmol/L MgCl_2_, 0.05% Tween-20, and 1 mmol/L DTT. ADP production was measured after a 100-minute incubation at room temperature, using the ADP-Glo kit (Promega, #V9102).

### Kinome Selectivity Profiling

*In vitro* kinase profiling assay of STX-478 was assessed across 373 kinases (KinaseProfiler, IC_50_Profiler; Eurofins Cerep).

### Cellular Assays

Cellular assay experiments were performed at a contract research organization (CRO) or internally at Scorpion Therapeutics. The BT-20 cell line was purchased by the CRO from ATCC (2016) and grown in DMEM with 10% FBS; it was last authenticated by short tandem repeat (STR) analysis and *Mycoplasma* tested in April 2023. The CAL-148 cell line was purchased by a CRO from DSMZ (2018) and grown in DMEM with 10% FBS; it was last authenticated by STR and *Mycoplasma* tested in May 2023. The CAL-33 cell line was purchased from DSMZ (2021) and last authenticated by STR and *Mycoplasma* tested in March 2022. CAL-33 was purchased by the CRO from CoBioer (2018); it was last authenticated by STR in September 2021 and *Mycoplasma* tested in June 2021. The Detroit 562 cell line was purchased from ATCC (2021) and grown in EMEM with 10% FBS; it was last authenticated by STR and *Mycoplasma* tested in August 2021. Detroit 562 was purchased by the CRO from ATCC (2015); it was last authenticated by STR in June 2023 and *Mycoplasma* tested in October 2022. The EFM-19 cell line was purchased from DSMZ (ACC 231) and grown in RPMI with 10% FBS; it was last authenticated by STR and *Mycoplasma* tested in December 2021. EFM-19 was purchased by the CRO from CoBioer (2018); it was last authenticated by STR in January 2022 and *Mycoplasma* tested in June 2023. The GP2d cell line was purchased from Sigma-Aldrich (2021) and grown in DMEM with 10% FBS; it was last authenticated by STR and *Mycoplasma* tested in August 2021. GP2d was purchased by the CRO from CoBioer; it was last authenticated by STR in October 2022 and *Mycoplasma* tested in December 2021. The HCC1954 cell line was purchased from ATCC (2020) and grown in RPMI with 10% FBS; it was last authenticated by STR in August 2021 and *Mycoplasma* tested in October 2021. HCC1954 was purchased by the CRO from ATCC (2016); it was last authenticated by STR in April 2023 and *Mycoplasma* tested in June 2023. The NCI-H1048 cell line was purchased from ATCC (2021) and grown in DMEM:F12 with 10% FBS; it was last authenticated by STR and *Mycoplasma* tested in August 2021. NCI-H1048 was purchased by the CRO from ATCC (2017); it was last authenticated by STR and *Mycoplasma* tested in March 2022. The OAW42 cell line was purchased by the CRO from CoBioer (2019) and grown in DMEM with 2 mmol/L glutamine, 1 mmol/L sodium pyruvate (NaP), 20 IU/L bovine insulin, and 10% FBS; it was last authenticated by STR and *Mycoplasma* tested in May 2023. The SKBR3 cell line was purchased from ATCC (2020) and grown in McCoy's with 10% FBS; it was last authenticated by STR in August 2020 and *Mycoplasma* tested in August 2021. SKBR3 was purchased by the CRO from ATCC (2011); it was authenticated by STR in January 2021 and *Mycoplasma* tested in May 2023. The T47D cell line was purchased from ATCC (2020) and grown in RPMI with 10% FBS and 0.2 units/mL bovine insulin; it was last authenticated by STR in December 2021 and *Mycoplasma* tested in May 2023. T47D was purchased by the CRO from ATCC (2015); it was last authenticated by STR in February 2023 and *Mycoplasma* tested in December 2021. The MCF10A isogenic cell lines (parent, H1047R± and E545K^+/−^) were purchased from Horizon Discovery (2020) and grown in Lonza's MEGM BulletKit media (#CC-3150) supplemented with 100 ng/mL cholera toxin. STR and *Mycoplasma* PCR tests were done at IDEXX; *Mycoplasma* testing done by the CRO was a biochemical test. Cells were not passaged more than 16 times internally and not more than 10 times at the CRO. See Supplementary Table S4 for additional details.

### pAKT Homogenous Time-Resolve Fluorescence

A 384-well phospho-AKT (S473) HTRF (Homogenous Time-Resolve Fluorescence; PerkinElmer, #64AKSPEH) assay was used for target engagement. Each well was seeded with cell numbers indicated in Supplementary Table S3 in a volume of 12.5 μL in complete, phenol red-free media and then treated for 1 hour at 37°C with 5% CO_2_ before reading on a PHERAstar plate reader.

### Cell Proliferation

Cell viability was measured using CellTiter-Glo (Promega, #G9243). Indicated cell lines were seeded according to Supplementary Table S3 in 50 μL of media in 384-well plates, with 72 hours of treatment at 37°C with 5% CO_2_.

STX-478 was submitted to the Broad Institute PRISM high-throughput cell viability screen (https://www.theprismlab.org/). The AUC metric was calculated using PharmacoGx R package (49).

### Human Adipocyte ^3^H-2-Deoxyglucose Uptake

Primary human subcutaneous adipocytes were treated with a test compound or vehicle for 1 hour followed by the addition of 10 nmol/L insulin and ^3^H-2-deoxyglucose (Zen Bio Durham, assay #CA-25, Lot #SL0071). Cytochalasin B treatment controlled for nonspecific glucose uptake. Corrected counts per minute were determined using a scintillation counter.

### Animal Studies

All animal handling and treatment procedures were performed according to the approved Institutional Animal Care and Use Committee guidelines following the Association for Assessment and Accreditation of Laboratory Animal Care guidance. Cell line xenografts were performed in BALB/c nude mice except for the T47D model that used NOD scid gamma immunodeficient (NSG) mice implanted with 17-beta estradiol tablets (0.5 mg, 90-day release). PK/PD studies with xenograft tumors were established using standard protocols. PDX models were performed by XenoSTART. STX-478 and alpelisib were formulated in 30% 2-hydroxylpropyl-beta-cyclodextrin pH 8. Tissue Western blots used standard protocols with snap-frozen tissues in radioimmunoprecipitation assay buffer with protease inhibitors. Primary antibodies pAKT (S473; AB_2315049), AKT (AB_1147620), and vinculin (AB_2728768) from Cell Signaling Technology, and secondary antibodies IRDye 680CW Goat anti-Mouse IgG (AB_10956588) and IRDye 800CW Goat anti-Rabbit IgG (AB_621843) from LI-COR were used. IHC samples were fixed in 10% NBF for 24 hours and transferred into 70% ethanol, followed by embedding, sectioning, staining, and quantification of tumor pAKT (Cell Signaling Technology #4060) and pS6 (Cell Signaling Technology #35708). Plasma and tissue bioanalytical analysis of STX-478 or alpelisib was carried out following protein precipitation using liquid chromatography with tandem mass spectrometry. All methods and limits of quantification were adequate regarding specificity and sensitivity to support the PK analysis.

OGTT and ITT studies in BALB/c nude were carried out after 5 days of treatment. Food was removed for 5 hours, drug administered, and then 2 g/kg oral glucose (OGTT), or 0.75 U/kg intraperitoneal insulin (ITT; Lilly, #H1079), was administered 1 hour later. Blood glucose was measured at times indicated following tail-vein collection or terminal bleed (One Touch Glucose Meter, Roche, ACCU-CHEK Performa, #06454038). Insulin was measured by ELISA (Crystal Chem, #90082). Metabolic profiling in CAL-33 tumor–bearing mice was initiated following a 4-hour fast with the dosing of the indicated drug. After 60 minutes, 300-mg ^13^C-labeled glucose (Cambridge Isotope Labs, #CLM-1396-0) was delivered by oral administration. Tissues were collected just prior to labeled glucose administration (0 hours) or after 30 minutes, snap-frozen, and analyzed (NYU metabolic core using Hybrid Metabolomics protocol, RRID:SCR_017935).

### Data Availability

Atomic coordinates and structure factors for the cocrystal X-ray structures of STX-478 with H1047R PI3Kα and WT PI3Kα have been deposited in the PDB with the codes 8TGD and 8TDU, respectively.

## Supplementary Material

Supplementary Tables and Figures Includes Supplementary Tables S1 - S4 and Supplementary Figures S1 - S6

## References

[1] Mayer IA , Arteaga CL. The PI3K/AKT pathway as a target for cancer treatment. Annu Rev Med 2016;67:11–28. 26473415 10.1146/annurev-med-062913-051343

[2] Fruman DA , Chiu H, Hopkins BD, Bagrodia S, Cantley LC, Abraham RT. The PI3K pathway in human disease. Cell 2017;170:605–35. 28802037 10.1016/j.cell.2017.07.029 PMC5726441

[3] Zhang Y , Kwok-Shing Ng P, Kucherlapati M, Chen F, Liu Y, Tsang YH, . A Pan-cancer proteogenomic atlas of PI3K/AKT/mTOR pathway alterations. Cancer Cell 2017;31:820–32. 28528867 10.1016/j.ccell.2017.04.013 PMC5502825

[4] Karakas B , Bachman KE, Park BH. Mutation of the PIK3CA oncogene in human cancers. Br J Cancer 2006;94:455–9. 16449998 10.1038/sj.bjc.6602970 PMC2361173

[5] Arafeh R , Samuels Y. PIK3CA in cancer: the past 30 years. Semin Cancer Biol 2019;59:36–49. 30742905 10.1016/j.semcancer.2019.02.002

[6] Belli C , Repetto M, Anand S, Porta C, Subbiah V, Curigliano G. The emerging role of PI3K inhibitors for solid tumour treatment and beyond. Br J Cancer 2023;128:2150–62. 36914722 10.1038/s41416-023-02221-1 PMC10241926

[7] Martinez-Saez O , Chic N, Pascual T, Adamo B, Vidal M, Gonzalez-Farre B, . Frequency and spectrum of PIK3CA somatic mutations in breast cancer. Breast Cancer Res 2020;22:45. 32404150 10.1186/s13058-020-01284-9 PMC7222307

[8] Cerami E , Gao J, Dogrusoz U, Gross BE, Sumer SO, Aksoy BA, . The cBio Cancer Genomics Portal: an open platform for exploring multidimensional cancer genomics data. Cancer Discov 2012;2:401–4. 22588877 10.1158/2159-8290.CD-12-0095 PMC3956037

[9] Gao J , Aksoy BA, Dogrusoz U, Dresdner G, Gross B, Sumer SO, . Integrative analysis of complex cancer genomics and clinical profiles using the cBioPortal. Sci Signal 2013;6:pl1. 23550210 10.1126/scisignal.2004088 PMC4160307

[10] Andre F , Ciruelos E, Rubovszky G, Campone M, Loibl S, Rugo HS, . Alpelisib for PIK3CA-mutated, hormone receptor-positive advanced breast cancer. N Engl J Med 2019;380:1929–40. 31091374 10.1056/NEJMoa1813904

[11] James DE , Stockli J, Birnbaum MJ. The aetiology and molecular landscape of insulin resistance. Nat Rev Mol Cell Biol 2021;22:751–71. 34285405 10.1038/s41580-021-00390-6

[12] Hanker AB , Kaklamani V, Arteaga CL. Challenges for the clinical development of PI3K inhibitors: strategies to improve their impact in solid tumors. Cancer Discov 2019;9:482–91. 30867161 10.1158/2159-8290.CD-18-1175 PMC6445714

[13] Rugo HS , Lacouture ME, Goncalves MD, Masharani U, Aapro MS, O'Shaughnessy JA. A multidisciplinary approach to optimizing care of patients treated with alpelisib. Breast 2022;61:156–67. 35016012 10.1016/j.breast.2021.12.016 PMC8749445

[14] Peairs KS , Barone BB, Snyder CF, Yeh HC, Stein KB, Derr RL, . Diabetes mellitus and breast cancer outcomes: a systematic review and meta-analysis. J Clin Oncol 2011;29:40–6. 21115865 10.1200/JCO.2009.27.3011 PMC3055858

[15] Cheung YM , Cromwell GE, Tolaney SM, Min L, McDonnell ME. Factors leading to alpelisib discontinuation in patients with hormone receptor positive, human epidermal growth factor receptor-2 negative breast cancer. Breast Cancer Res Treat 2022;192:303–11. 35000092 10.1007/s10549-021-06476-1

[16] Bello Roufai D , Gonçalves A, De La Motte Rouge T, Akla S, Blonz C, Grenier J, . Alpelisib and fulvestrant in PIK3CA-mutated hormone receptor-positive HER2-negative advanced breast cancer included in the French early access program. Oncogene 2023;2:1951–6. 10.1038/s41388-022-02585-3 36611120

[17] Hopkins BD , Pauli C, Du X, Wang DG, Li X, Wu D, . Suppression of insulin feedback enhances the efficacy of PI3K inhibitors. Nature 2018;560:499–503. 30051890 10.1038/s41586-018-0343-4 PMC6197057

[18] Rugo HS , Andre F, Yamashita T, Cerda H, Toledano I, Stemmer SM, . Time course and management of key adverse events during the randomized phase III SOLAR-1 study of PI3K inhibitor alpelisib plus fulvestrant in patients with HR-positive advanced breast cancer. Ann Oncol 2020;31:1001–10. 32416251 10.1016/j.annonc.2020.05.001

[19] Wang DG , Barrios DM, Blinder VS, Bromberg JF, Drullinsky PR, Funt SA, . Dermatologic adverse events related to the PI3Kα inhibitor alpelisib (BYL719) in patients with breast cancer. Breast Cancer Res Treat 2020;183:227–37. 32613539 10.1007/s10549-020-05726-y PMC7398571

[20] Vasan N , Cantley LC. At a crossroads: how to translate the roles of PI3K in oncogenic and metabolic signalling into improvements in cancer therapy. Nat Rev Clin Oncol 2022;19:471–85. 35484287 10.1038/s41571-022-00633-1 PMC11215755

[21] Fritsch C , Huang A, Chatenay-Rivauday C, Schnell C, Reddy A, Liu M, . Characterization of the novel and specific PI3Kα inhibitor NVP-BYL719 and development of the patient stratification strategy for clinical trials. Mol Cancer Ther 2014;13:1117–29. 24608574 10.1158/1535-7163.MCT-13-0865

[22] Mandelker D , Gabelli SB, Schmidt-Kittler O, Zhu J, Cheong I, Huang CH, . A frequent kinase domain mutation that changes the interaction between PI3Kα and the membrane. Proc Natl Acad Sci U S A 2009;106:16996–7001. 19805105 10.1073/pnas.0908444106 PMC2761334

[23] Elkabets M , Vora S, Juric D, Morse N, Mino-Kenudson M, Muranen T, . mTORC1 inhibition is required for sensitivity to PI3K p110α inhibitors in PIK3CA-mutant breast cancer. Sci Transl Med 2013;5:196ra99. 10.1126/scitranslmed.3005747 PMC3935768 23903756

[24] Razavi P , Dickler MN, Shah PD, Toy W, Brown DN, Won HH, . Alterations in PTEN and ESR1 promote clinical resistance to alpelisib plus aromatase inhibitors. Nat Cancer 2020;1:382–93. 32864625 10.1038/s43018-020-0047-1 PMC7450824

[25] Hauner H , Rohrig K, Spelleken M, Liu LS, Eckel J. Development of insulin-responsive glucose uptake and GLUT4 expression in differentiating human adipocyte precursor cells. Int J Obes Relat Metab Disord 1998;22:448–53. 9622342 10.1038/sj.ijo.0800606

[26] Juric D , Rodon J, Tabernero J, Janku F, Burris HA, Schellens JHM, . Phosphatidylinositol 3-kinase alpha-selective inhibition with alpelisib (BYL719) in PIK3CA-altered solid tumors: results from the first-in-human study. J Clin Oncol 2018;36:1291–9. 29401002 10.1200/JCO.2017.72.7107 PMC5920739

[27] Dockx Y , Vangestel C, Van den Wyngaert T, Huizing M, De Bruycker S, Pauwels P, . Early changes in [^18^F]FDG uptake as a readout for PI3K/Akt/mTOR targeted drugs in HER-2-positive cancer xenografts. Mol Imaging 2021;2021:5594514. 34113218 10.1155/2021/5594514 PMC8169268

[28] Sarker D , Ang JE, Baird R, Kristeleit R, Shah K, Moreno V, . First-in-human phase I study of pictilisib (GDC-0941), a potent pan-class I phosphatidylinositol-3-kinase (PI3K) inhibitor, in patients with advanced solid tumors. Clin Cancer Res 2015;21:77–86. 25370471 10.1158/1078-0432.CCR-14-0947 PMC4287394

[29] Lopes M , Brejchova K, Riecan M, Novakova M, Rossmeisl M, Cajka T, . Metabolomics atlas of oral 13C-glucose tolerance test in mice. Cell Rep 2021;37:109833. 34644567 10.1016/j.celrep.2021.109833

[30] Serra V , Scaltriti M, Prudkin L, Eichhorn PJ, Ibrahim YH, Chandarlapaty S, . PI3K inhibition results in enhanced HER signaling and acquired ERK dependency in HER2-overexpressing breast cancer. Oncogene 2011;30:2547–57. 21278786 10.1038/onc.2010.626 PMC3107390

[31] Chandarlapaty S , Sawai A, Scaltriti M, Rodrik-Outmezguine V, Grbovic-Huezo O, Serra V, . AKT inhibition relieves feedback suppression of receptor tyrosine kinase expression and activity. Cancer Cell 2011;19:58–71. 21215704 10.1016/j.ccr.2010.10.031 PMC3025058

[32] Savas P , Lo LL, Luen SJ, Blackley EF, Callahan J, Moodie K, . Alpelisib monotherapy for PI3K-altered, pretreated advanced breast cancer: a phase II study. Cancer Discov 2022;12:2058–73. 35771551 10.1158/2159-8290.CD-21-1696

[33] Juric D , Janku F, Rodon J, Burris HA, Mayer IA, Schuler M, . Alpelisib plus fulvestrant in PIK3CA-altered and PIK3CA-wild-type estrogen receptor-positive advanced breast cancer: a phase 1b clinical trial. JAMA Oncol 2019;5:e184475. 30543347 10.1001/jamaoncol.2018.4475 PMC6439561

[34] O'Brien NA , McDermott MSJ, Conklin D, Luo T, Ayala R, Salgar S, . Targeting activated PI3K/mTOR signaling overcomes acquired resistance to CDK4/6-based therapies in preclinical models of hormone receptor-positive breast cancer. Breast Cancer Res 2020;22:89. 32795346 10.1186/s13058-020-01320-8 PMC7427086

[35] Rosfjord E , Lucas J, Li G, Gerber HP. Advances in patient-derived tumor xenografts: from target identification to predicting clinical response rates in oncology. Biochem Pharmacol 2014;91:135–43. 24950467 10.1016/j.bcp.2014.06.008

[36] Castel P , Toska E, Engelman JA, Scaltriti M. The present and future of PI3K inhibitors for cancer therapy. Nat Cancer 2021;2:587–97. 35118422 10.1038/s43018-021-00218-4 PMC8809509

[37] Vanhaesebroeck B , Perry MWD, Brown JR, Andre F, Okkenhaug K. PI3K inhibitors are finally coming of age. Nat Rev Drug Discov 2021;20:741–69. 34127844 10.1038/s41573-021-00209-1 PMC9297732

[38] Gkeka P , Papafotika A, Christoforidis S, Cournia Z. Exploring a non-ATP pocket for potential allosteric modulation of PI3Kalpha. J Phys Chem B 2015;119:1002–16. 25299356 10.1021/jp506423e

[39] Sivakumar S , Jin DX, Rathod R, Ross J, Cantley LC, Scaltriti M, . Genetic heterogeneity and tissue-specific patterns of tumors with multiple PIK3CA mutations. Clin Cancer Res 2023;29:1125–36. 36595567 10.1158/1078-0432.CCR-22-2270 PMC10011881

[40] Tolaney SM , Im YH, Calvo E, Lu YS, Hamilton E, Forero-Torres A, . Phase Ib study of ribociclib plus fulvestrant and ribociclib plus fulvestrant plus PI3K inhibitor (alpelisib or buparlisib) for HR(+) advanced breast cancer. Clin Cancer Res 2021;27:418–28. 32887722 10.1158/1078-0432.CCR-20-0645

[41] Buckbinder L , St. Jean DJ, Ladd B, Tieu T, Jonsson P, Alltucker J, . STX-478, a mutant-selective PI3Kα H1047X inhibitor clinical candidate with a best-in-class profile: pharmacology and therapeutic activity as monotherapy and in combination in breast cancer xenograft models [abstract]. In: Proceedings of the 2022 San Antonio Breast Cancer Symposium; 2022 Dec 6–10; San Antonio, TX. Philadelphia (PA): AACR; Cancer Res 2023;83(5 Suppl):Abstract nr P4-07-04.

[42] Burke JE , Perisic O, Masson GR, Vadas O, Williams RL. Oncogenic mutations mimic and enhance dynamic events in the natural activation of phosphoinositide 3-kinase p110alpha (PIK3CA). Proc Natl Acad Sci U S A 2012;109:15259–64. 22949682 10.1073/pnas.1205508109 PMC3458343

[43] Zhao L , Vogt PK. Class I PI3K in oncogenic cellular transformation. Oncogene 2008;27:5486–96. 18794883 10.1038/onc.2008.244 PMC2757120

[44] Madsen RR , Vanhaesebroeck B, Semple RK. Cancer-associated PIK3CA mutations in overgrowth disorders. Trends Mol Med 2018;24:856–70. 30197175 10.1016/j.molmed.2018.08.003 PMC6185869

[45] St. Jean DJ Jr , Cummings MD, inventors. Urea derivatives which can be used to treat cancer. World Intellectual Property Organization patent WO2022265993. 2022 Dec 12.

[46] Kabsch W . Integration, scaling, space-group assignment and post-refinement. Acta Crystallogr D Biol Crystallogr 2010;66( Pt 2):133–44. 20124693 10.1107/S0907444909047374 PMC2815666

[47] Evans P . Scaling and assessment of data quality. Acta Crystallogr D Biol Crystallogr 2006;62( Pt 1):72–82. 16369096 10.1107/S0907444905036693

[48] Vonrhein C , Flensburg C, Keller P, Sharff A, Smart O, Paciorek W, . Data processing and analysis with the autoPROC toolbox. Acta Crystallogr D Biol Crystallogr 2011;67( Pt 4):293–302. 21460447 10.1107/S0907444911007773 PMC3069744

[49] Smirnov P , Safikhani Z, El-Hachem N, Wang D, She A, Olsen C, . PharmacoGx: an R package for analysis of large pharmacogenomic data sets. Bioinformatics 2016;32:1244–6. 26656004 10.1093/bioinformatics/btv723

