# Supplementary material for: STX-478, a Mutant-Selective, Allosteric PI3Kα Inhibitor Spares Metabolic Dysfunction and Improves Therapeutic Response in PI3Kα-Mutant Xenografts
Source: Cancer Discov. 2023 Aug 25;13(11):2432–47. doi: 10.1158/2159-8290.CD-23-0396 (PMC10618743; doi:10.1158/2159-8290.CD-23-0396)
Supplement: Supplementary Tables and Figures — Includes Supplementary Tables S1 - S4 and Supplementary Figures S1 - S6 [file cd-23-0396_supplementary_tables_and_figures_suppsd1.pdf]

Structure	H1047R / STX-478	WT / STX-478
**PDB Entry**	**8TGD**	**8TDU**
**Data collection**		
Resolution (Å)	34.42–2.93 (3.34–2.93)	48.77-3.11 (3.19-3.11)
Space group	$P2_1$	$P2_1$
Cell dimensions		
a, b, c, (Å)	86.2, 124.5, 165.4	86.3, 124.7, 165.8
α, β, γ (°)	90.0, 92.9, 90.0	90.0, 92.8, 90.0
No. unique reflections	55,994 (5600)	63223 (4400)
I/σ(I)	11.5 (1.7)	14.4 (2.3)
Completeness (%)	94.1 (65.3)	100.0 (99.9)
Multiplicity	4.8 (5.0)	6.9 (7.1)
R_{meas}	0.11 (1.10)	0.10 (0.91)
R_{pim}	0.05 (0.49)	0.04 (0.34)
CC(1/2)	0.998 (0.582)	0.999 (0.786)
**Refinement**		
R_{work}	0.218 (0.308)	0.210 (0.313)
R_{free}	0.252 (0.377)	0.265 (0.376)
RMS deviations		
Bond lengths (Å)	0.008	0.003
Bond angles (°)	0.89	0.97

Supplementary Table S1. STX-478 X-ray crystallography statistics. Values in parentheses represent the highest resolution shell. RMS, root mean square.

Cell line	Genetic PI3Kα status	RRID	STX-478 pAKT HTRF GMean IC_{50} (nM) (95% CI)	STX-478 CTGlo GMean IC_{50} (nM) (95% CI)	Alpelisib pAKT HTRF GMean GI_{50} (nM) (95% CI)	Alpelisib CTGlo GMean GI_{50} (nM) (95% CI)
BT20	H1047R + P539R	CVCL_0178	46 (27–79)	654 (547–783)	136 (58–321)	516 (435–612)
CAL148	H1047R + D350N	CVCL_1106	121 (106–139)	705 (555–895)	268 (166–431)	962 (774–1196)
CAL33	H1047R	CVCL_1108	79 (67–92)	577 (468–711)	71 (65–77)	340 (301–383)
DETROIT562	H1047R	CVCL_1171	88 (63–123)	246 (181–333)	125 (79–199)	436 (336–565)
EFM19	H1047L	CVCL_0253	15 (13–16)	20 (16–25)	43 (37–50)	127 (110–147)
GP2D	H1047L	CVCL_2450	28 (20–37)	82 (53–127)	66 (49–89)	298 (218–408)
HCC1954	H1047R	CVCL_1259	211 (177–252)	457 (300–697)	256 (210–313)	384 (264–560)
NCIH1048	H1047R + K111R	CVCL_1453	18 (15–22)	45 (38–53)	28 (19–43)	67 (48–94)
OAW42	H1047L	CVCL_1615	28 (20–40)	430 (342–541)	82 (44–155)	601 (529–682)
SKBR3	WT	CVCL_0033	319 (290–351)	1559 (1265–1921)	41 (38-44)	335 (285–394)
T47D	H1047R	CVCL_0553	36 (31–42)	116 (88–151)	58 (54–62)	334 (299–372)

Supplementary Table S2. STX-478 target engagement and cell viability activity in a human tumor cell line panel. GI_{50}, concentration of compound that reduces total cell growth by 50%; GMean, geometric mean; RRID, Research Resource Identifier.

Cell line (day of final dose)	Cancer type	PI3Kα mutation	STX-478 100 mg/kg QD		Alpelisib 50 mg/kg QD	
			TGI (%)[a]	Δ Insulin[b] (ng/mL)	TGI (%)[a]	Δ Insulin[b] (ng/mL)
Cal33 (Day 28)	HNSCC	H1047R	82****	+0.17[NS]	79****	+1.07[NS]
Detroit 562 (Day 22)	HNSCC	H1047R	92****	+0.21[NS]	96****	+2.33**
GP2D (Day 28)	Colon	H1047L	-2****	+0.03[NS]	79****	+1.59****
NCI-H1048 (Day 23)	Lung	H1047R + K111R	85****	+0.37[NS]	87****	+1.21**
HCC1954 (Day 28)	Breast	H1047R	69*	+0.33[NS]	77**	+2.38**
T-47D[d] (Day 20)	Breast	H1047R	-52****	+0.28[NS]	100****	+0.79****

TGI or [a]regression (negative TGI) relative to Day 1 dosing.
[b]One-hour post dose.
$P \leq$ 0.05*, 0.01**, 0.001***, 0.0001****.

<u>Supplementary Table S3.</u> Summary of CDX studies: TGI and insulin levels (1-hour post-dose). Negative TGI values indicate % regression. NS, not significant.

Cell Line	RRID	HTRF seeding density (384 well)	CTGlo seeding density (384 well)
T47D	CVCL_0178	5000	1500
CAL-33	CVCL_1106	5000	1200
SKBR3	CVCL_1108	5000	1500
NCI-H1048	CVCL_1171	10,000	1500
HCC1954	CVCL_0253	5000	2000
OAW42	CVCL_2450	5000	500
Detroit562	CVCL_1259	5000	500
BT-20	CVCL_1453	5000	1000
GP2d	CVCL_1615	5000	1000
CAL-148	CVCL_0553	5000	1000
EFM-19	CVCL_0033	5000	3000
MCF10A	CVCL_0598	3000	--
MCF10A [H1047R+/-]	CVCL_LD55	3000	--
MCF10A [E545K+/-]	CVCL_LD75	3000	--

Supplementary Table S4. **Cell lines and plating density used for HTRF (pAKT) and viability (CTGlo).**

A

Kinase	Alpelisib IC_{50} (nM)	STX-478 IC_{50} (nM)
PI3Kβ	>1000	50,000
PI3Kγ	250	>100,000
PI3Kδ	290	9700
Kinome profiling	11/254 kinases <10,000	1/373 kinases* <10,000

B

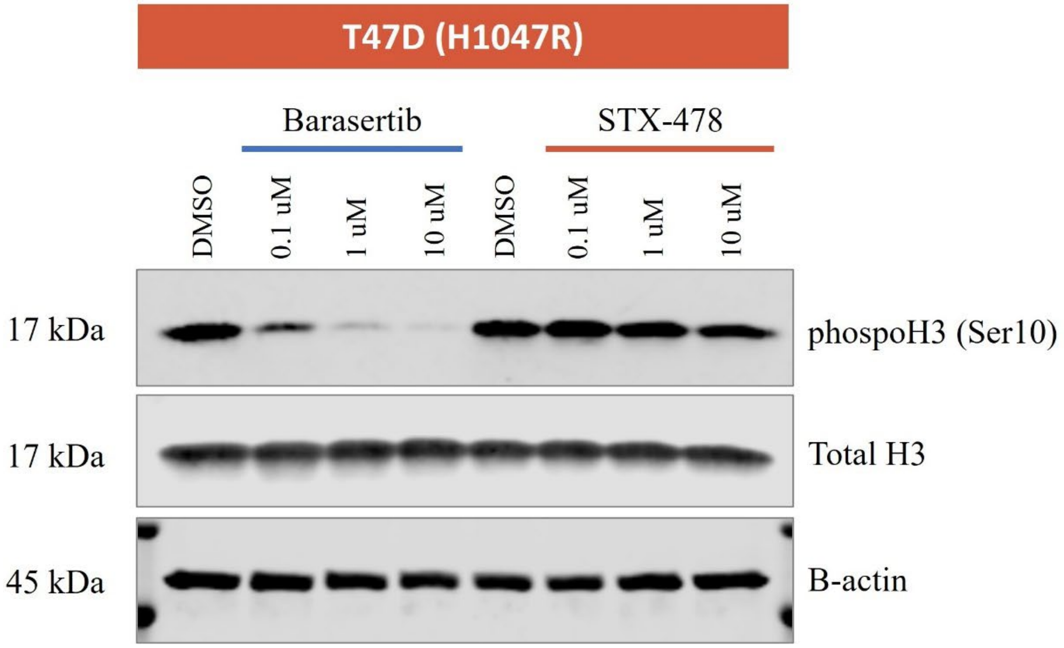

<u>**Supplementary Figure S1.**</u> **STX-478 has broad kinome selectivity. A,** Table summarizing STX-478 kinome profiling compared with published alpelisib data (21). AurB kinase as the only off-target with an IC_{50} < 10 μM (1658 nM). **B,** Dose-dependent inhibition of phospho-H3 (Ser10) observed 1-hour post treatment with AurB kinase inhibitor, barasertib. There was no significant inhibition of AurB observed with STX-478 treatment.

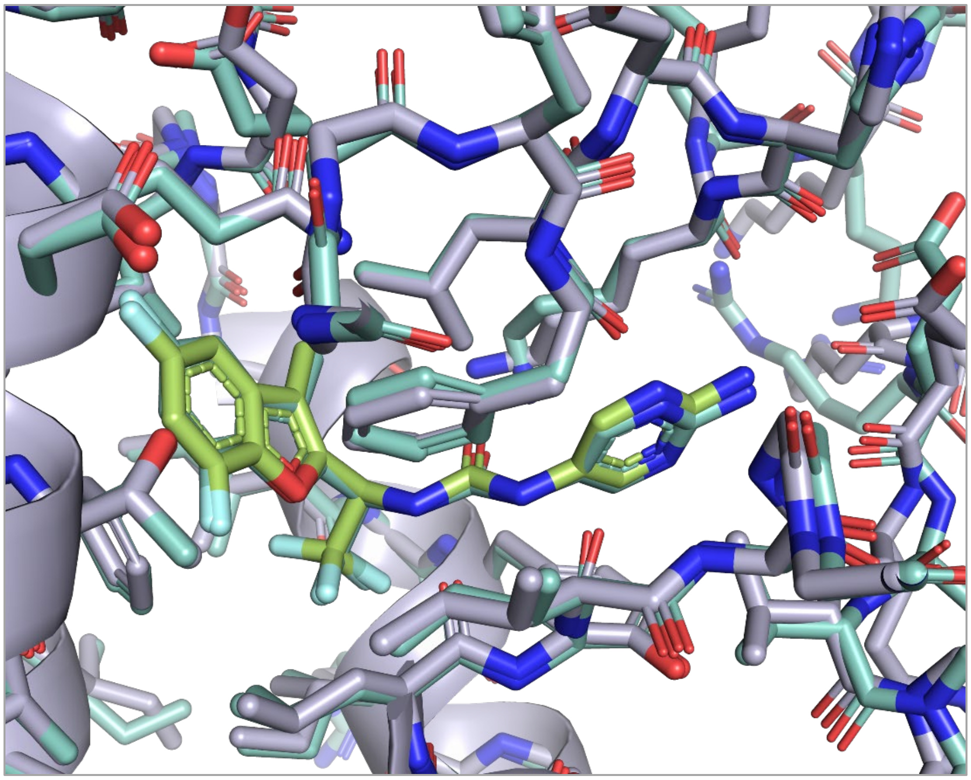

<u>Supplementary Figure S2.</u> The H1047R and WT STX-478-bound allosteric sites have the same conformation. The WT p110 / STX-478 structure (teal) aligns well with H1047R p110 (purple) / STX-478 (green). There are no significant differences in protein conformation and STX-478 contacts within the allosteric site, suggesting that mutant selectivity is not structure-driven.

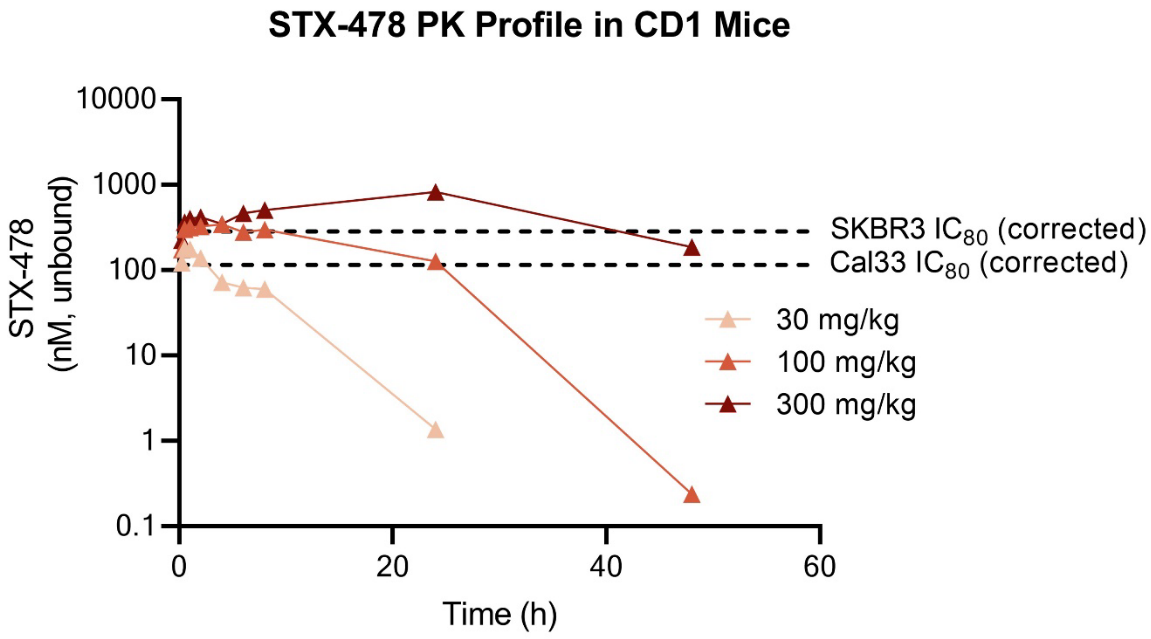

Supplementary Figure S3. STX-478 PK. Exposure versus time relationship following a single oral dose of STX-478 30, 100, or 300 mg/kg in CD1 mice. Total exposure and IC_{80} potency are corrected for both mouse PPB (fu = 0.033) and assay media binding (10% FBS fu = 0.26), respectively.

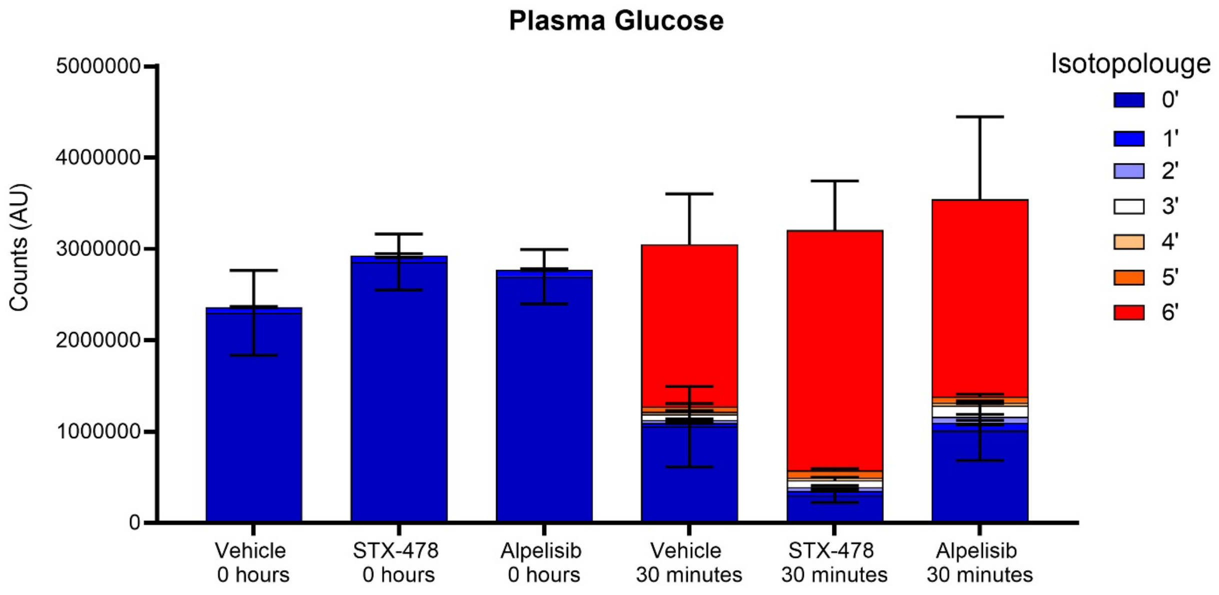

<u>**Supplementary Figure S4.**</u> **[U-^{13}C]-glucose labeling in plasma.** Plasma from mice represented in Fig. 4H. The isotopolouge state represents the counts of labeled U-^{13}C carbons on glucose. Animals from the 0-hour group were not dosed with glucose; samples were collected at the time glucose was administered to the mice collected 30-minutes post [U-^{13}C]-glucose. Bars represent SD.

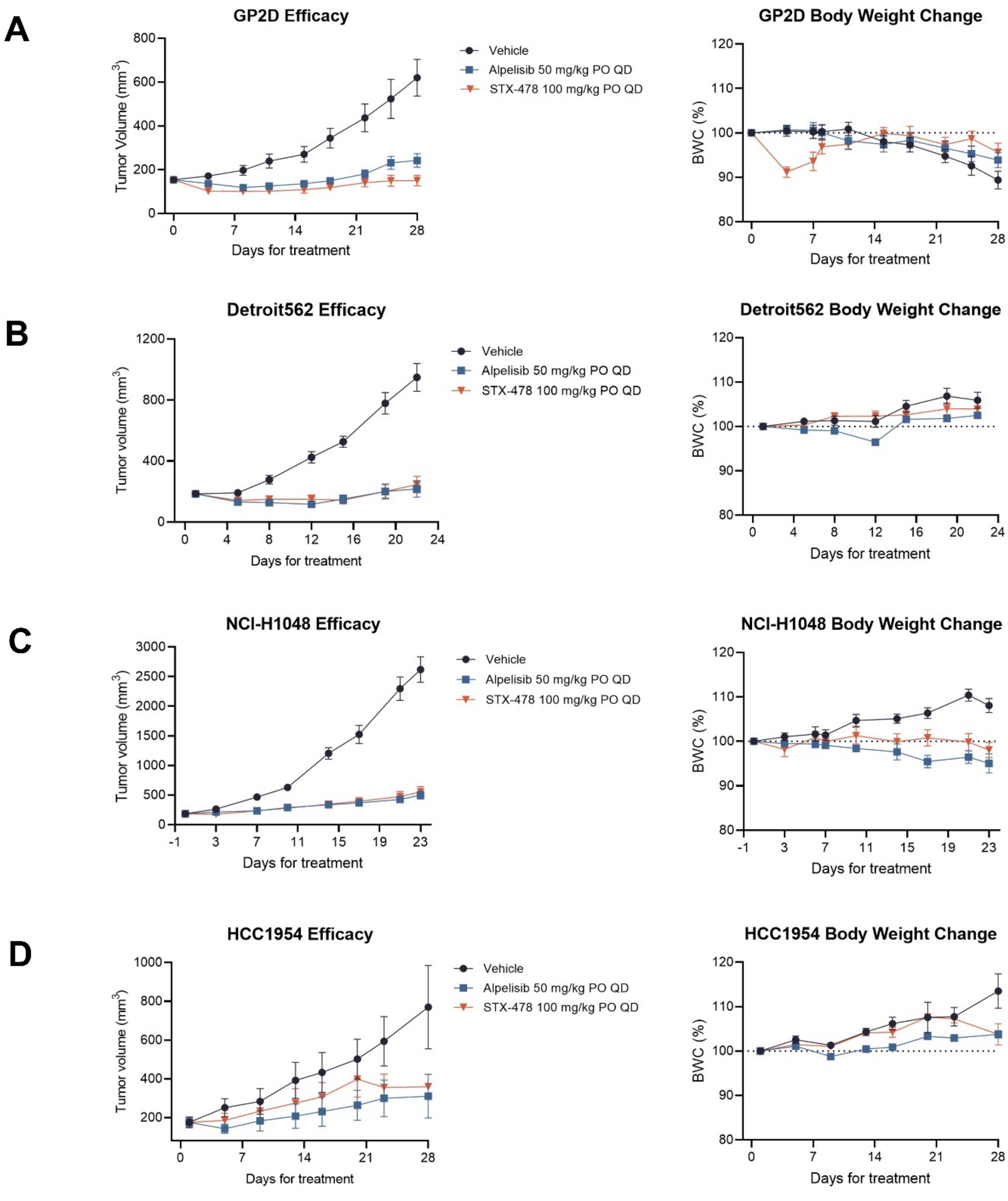

<u>**Supplementary Figure S5.**</u> *Continued on next page.*

E

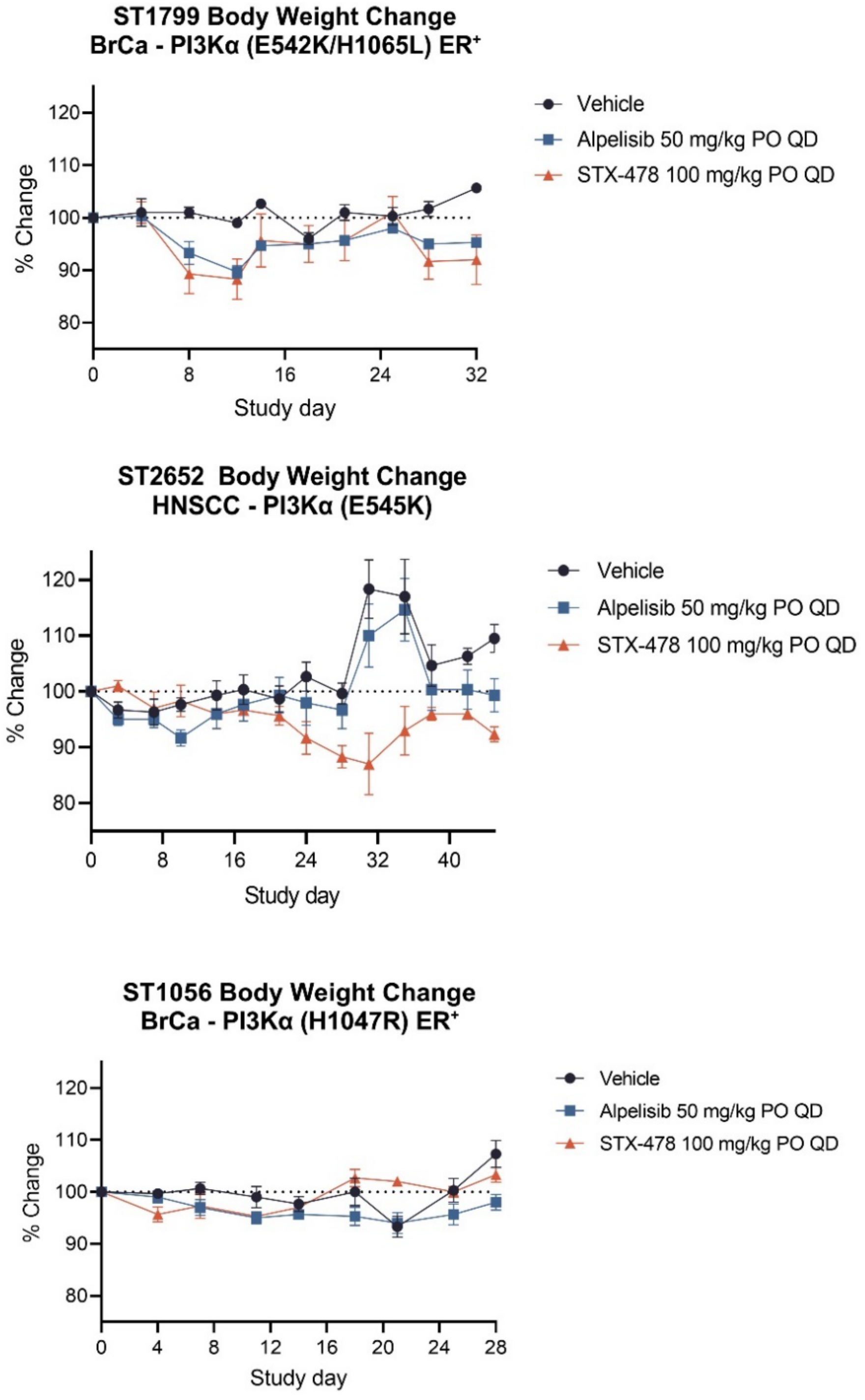

<u>**Supplementary Figure S5.**</u> *Continued on next page.*

F

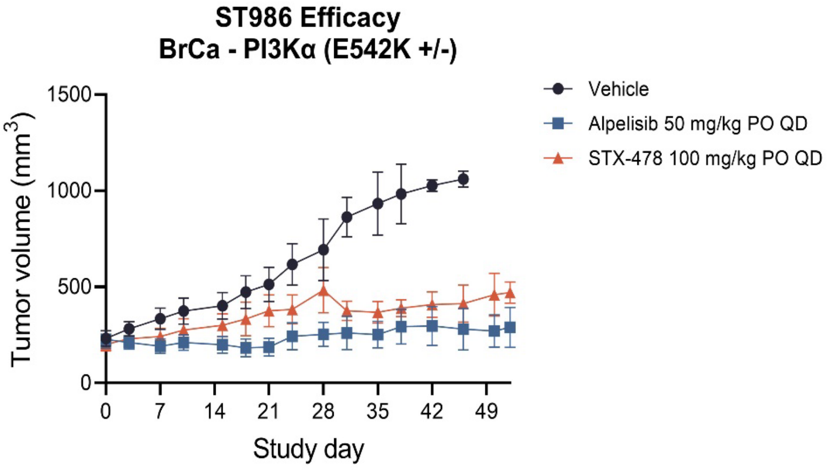

Supplementary Figure S5. Efficacy and tolerability of STX-478 in CDX and PDX models. **A–D,** Tumor volume measurements (left) and percent change in body weight (right) over time from CDX efficacy studies represented in Fig. 5A. N = 9 for NCI-H1048, Detroit562, and GP2D models; n = 6 for HCC1954. **E,** Percent change in body weight changes in PDX efficacy studies represented in Fig 5B. N = 3 in each model; bars represent SEM. **F,** Efficacy of STX-478 in ST986 PDX model. N = 3; bars represent SEM.

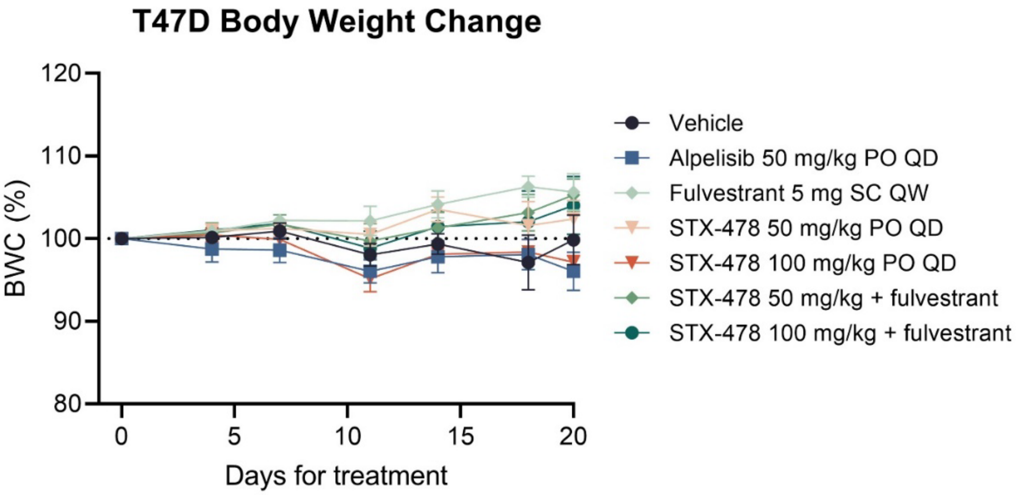

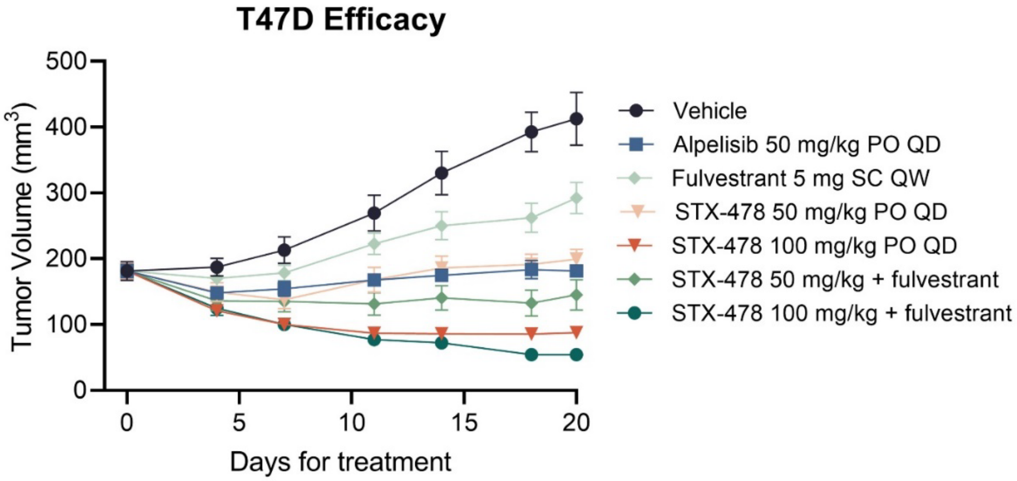

Supplementary Figure S6. Percent change in body weight (top) and tumor volume measurements (bottom) over time from the T47D efficacy study represented in Fig. 6A. N = 9.
